# Targeted sequencing reveals clonal genetic changes in the progression of early lung neoplasms and paired circulating DNA

**DOI:** 10.1038/ncomms9258

**Published:** 2015-09-16

**Authors:** Evgeny Izumchenko, Xiaofei Chang, Mariana Brait, Elana Fertig, Luciane T. Kagohara, Atul Bedi, Luigi Marchionni, Nishant Agrawal, Rajani Ravi, Sian Jones, Mohammad O. Hoque, William H. Westra, David Sidransky

**Affiliations:** 1 Department of Otolaryngology and Head and Neck Surgery, Johns Hopkins University School of Medicine, Baltimore, Maryland 21231, USA; 2 Division of Biostatistics and Bioinformatics, Johns Hopkins University School of Medicine, Baltimore, Maryland 21205, USA; 3 Center for Computational Genomics, Johns Hopkins University School of Medicine, Baltimore, Maryland 21231, USA; 4 Personal Genome Diagnostics, Inc., 2809 Boston Street, Suite 503, Baltimore, Maryland 21224, USA; 5 Department of Pathology, Johns Hopkins Medical Institutions, Baltimore, Maryland 21231, USA; 6 Department of Oncology, Johns Hopkins University School of Medicine, Baltimore, Maryland 21231, USA

## Abstract

Lungs resected for adenocarcinomas often harbour minute discrete foci of cytologically atypical pneumocyte proliferations designated as atypical adenomatous hyperplasia (AAH). Evidence suggests that AAH represents an initial step in the progression to adenocarcinoma *in situ* (AIS), minimally invasive adenocarcinoma (MIA) and fully invasive adenocarcinoma. Despite efforts to identify predictive markers of malignant transformation, alterations driving this progression are poorly understood. Here we perform targeted next-generation sequencing on multifocal AAHs and different zones of histologic progression within AISs and MIAs. Multiregion sequencing demonstrated different genetic drivers within the same tumour and reveal that clonal expansion is an early event of tumorigenesis. We find that *KRAS*, *TP53* and *EGFR* mutations are indicators of malignant transition. Utilizing droplet digital PCR, we find alterations associated with early neoplasms in paired circulating DNA. This study provides insight into the heterogeneity of clonal events in the progression of early lung neoplasia and demonstrates that these events can be detected even before neoplasms have invaded and acquired malignant potential.

## Abstract (web summary)

Atypical adenomatous hyperplasia is thought to be a precursor lesion for lung adenocarcinoma. Here, using targeted deep sequencing, the authors demonstrate that hyperplastic lesions contain somatic mutations associated with malignant disease and that these can be detected in circulating tumour cells.

## Graphical abstract

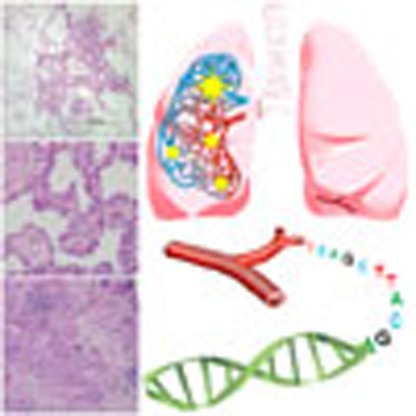

Adenocarcinomas represent the most frequent subtype of lung cancer^1^, and they are usually discovered late in the course of the disease even in the setting of vigilant radiographic and cytologic screening^2^. Despite improvements in molecular diagnosis and targeted therapies, the average 5 year survival rate for lung adenocarcinoma remains only 15% (ref. 3). Novel strategies based on the detection of genetic markers offer new hope for improved risk assessment, early cancer detection, therapeutic intervention and tumour surveillance, but the impact of these strategies has been limited by an incomplete understanding of the biology of lung cancer, particularly in its early developmental stages. Disappointingly, relatively few genetic alterations critical to the development of lung adenocarcinomas are currently recognized, and the timing and manner by which these alterations initiate and drive glandular neoplasia remains to be delineated.

Recent refinements in the histologic classification of lung adenocarcinomas provide greater resolution of the sequential steps of glandular lung neoplasia^4^. Atypical adenomatous hyperplasia (AAH) is a microscopic discrete focus of cytologically atypical type II pneumocytes and/or Clara cells^5^,^6^,^7^. Once dismissed as a reactive change, AAH is now regarded as the first histologic step in a morphologic continuum culminating in the fully malignant adenocarcinoma. The link between AAH and invasive adenocarcinoma is strong and compelling: 5–20% of lungs resected for primary adenocarcinomas also harbour AAH, and AAH harbours some of the same genetic and epigenetic alterations found in adenocarcinomas including *KRAS* mutations^8^, *EGFR* mutations^9^,^10^, loss of heterozygosity at 9q and 16p (ref. 11), *TP53* mutations^12^, and epigenetic alterations in the WNT pathway^13^. Like AAH, AIS (formerly known as bronchioloalveolar carcinoma, BAC) is recognized as a non-invasive form of glandular neoplasia, but one that exhibits increased size, cellularity and morphologic atypia. In effect, it represents a next step in the continuum towards malignant adenocarcinoma. Minimally invasive adenocarcinoma (MIA) is defined as a small adenocarcinoma (≤3 cm) with a predominantly lepidic pattern and invasion of 5 mm or less in any one focus^4^. Invasive growth is present, albeit so limited that these carcinomas have been associated with 100% disease free survival^14^,^15^,^16^. This enhanced delineation of early glandular neoplasia provides a rational histologic framework for studying the timing of genetic alterations driving the early stages of lung tumorigenesis.

‘Branched evolutionary tumour growth’ is the concept that cancers evolve by a repetitive process of clonal expansion, genetic diversification and clonal selection within the adaptive landscapes of tissue ecosystems^17^. In this study, to determine whether this phenomenon is operational during early stages of tumour progression, we evaluated lung glandular neoplasms spanning the full spectrum of early histologic progression using next-generation sequencing (NGS) of coding regions from 125 well-characterized cancer-driving genes. We specifically targeted multifocal AAHs and advancing zones of histologic progression within individual AISs and MIAs. This multiregion sequencing revealed that clonal expansion is an early event that can be confirmed even in the earliest recognized step in glandular neoplasia. Moreover, the identification of significant genetic alterations such as *KRAS* mutations, loss of P53 activity and EGFR activation points to the presence of functionally relevant ‘drivers’ that empower territorial expansion of subclones *en route* to malignancy. Importantly, these driver alterations are potentially measurable in clinical samples. Using ultra-sensitive droplet digital PCR (ddPCR), mutant DNA associated with early lesions was detected in a patient’s plasma and sputum providing proof of principle that even the earliest stages of glandular neoplasia can be detected via analysis of circulating DNA (circDNA).

## Results

### Mutational landscape in AAH

**Figure 1:**
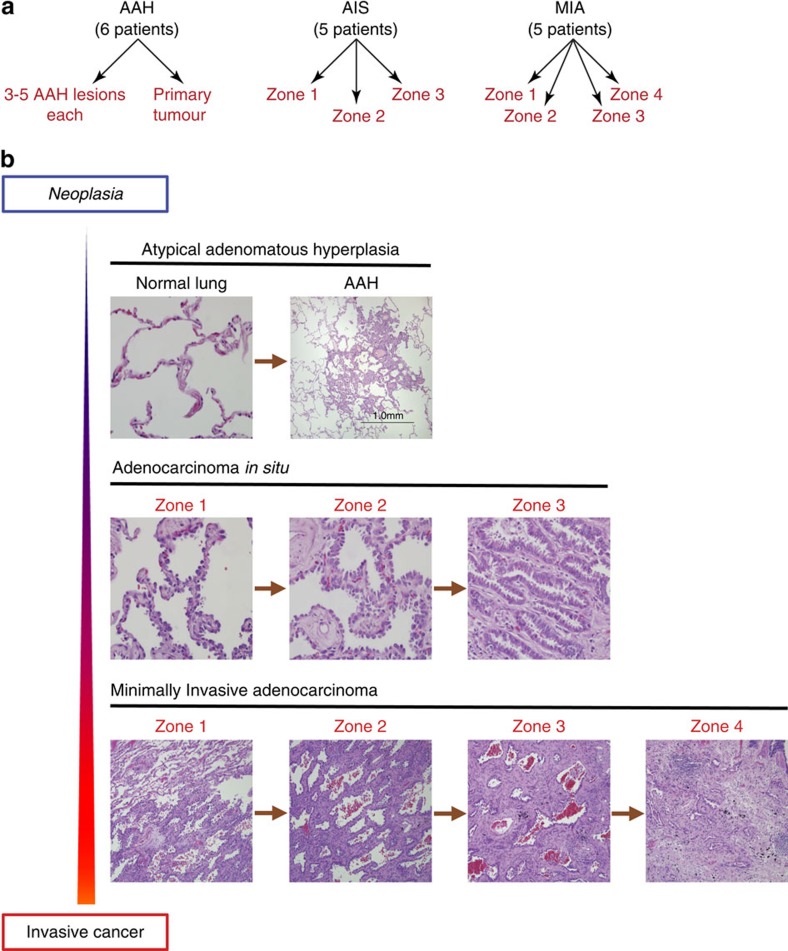
Histological characteristics of samples. (**a**) DNA was extracted from multiple AAH lesions collected from six patients on resection of primary lung adenocarcinoma. Samples from AIS and MIA tumours extracted from five patients each were collected from different zones of histologic progression within the same lesion. (**b**) AAH is the earliest form of glandular neoplasia of the lung, which characterized by the proliferation of slightly atypical epithelial cells lining the slightly thickened by intact septae. Size is a critical feature in distinguishing AAH from AIS, a scale bar (1.0 mm) was included to underscore the small size of the AAH (magnification × 4). AIS is characterized by atypical cells with enlarged hyperchromatic nuclei lining intact alveolated lung parenchyma (zone 1). With progression towards the centre of the tumour, the septae become increasingly thickened and the cytologic atypia becomes more pronounced (zone 2 and zone 3) (magnification × 40 for normal lung and all AIS panels). MIA exhibits increasing septal thickening and epithelial atypia with progression from the periphery of the lesion (zone 1) towards its centre (zone 2 and zone 3). In addition, there is a small focus (<0.5 cm) of invasion at the core of the lesion (zone 4) characterized by irregular acinar glands in a desmoplastic stroma (magnification × 10 for all MIA panels).

To explore heterogeneity in early and pre-neoplastic lesions in the bronchial tree, we first sequenced 25 distinct AAHs incidentally discovered in the lung resection specimens from six patients with invasive adenocarcinoma (Fig. 1a,b). We used a *CancerSelect* genes panel designed by Personal Genome Diagnostics. This panel consists of 125 well-characterized drivers common to many solid tumour types, including the clinically actionable genes in Non-small cell lung carcinoma (NSCLC) (Methods) (Supplementary Table 1).

Sequencing of AAHs and normal DNA to mean high-quality coverage depths of 415 × and 403 × , respectively (Supplementary Table 2), identified 56 non-synonymous alterations with an average mutation rate of 2.2 mutations per lesion (range 0–6 mutations per AAH; Table 1; Supplementary Table 3). Fourteen codon specific mutations were previously reported in various hematopoietic and solid tumours including lung adenocarcinomas (Supplementary Table 4), supporting their status as legitimate somatic mutations with potential functional implications. The analysis also disclosed mutations in genes rarely associated with lung adenocarcinoma (Supplementary Table 5). Only a fraction of affected genes were mutated in more than one patient (Table 1). The most frequently mutated genes were *BRAF* and *ARID1B*, mutated in four (16%) AAHs each, derived from four and two different patients, respectively (Supplementary Table 6). *EGFR* and *MAML1* were mutated in three AAHs in two of six patients each, while *TP53* and *KRAS* were mutated in several AAHs within the same lung. Alterations in growth factor receptors (others than EGFR) were seen in seven (28%) AAHs across five patients (Table 1). Like *EGFR*, these genes play role in regulation of AKT signalling^18^ and may contribute to survival via inhibition of apoptosis and induction of cell proliferation^19^. Notably, genes commonly associated with DNA repair and chromatin remodelling network, such as *ATM*^20^, *ASXL1* (ref. 21), *ATRX*^22^, *EZH2* (ref. 23), *ARID1A*^24^ and *ARID1B*^24^, were mutated in multiple AAHs in four of six patients (Table 1), suggesting the predisposition of these lesions to the acquisition of the secondary genetic abnormalities.

### Mutational landscape in AIS and MIA tumours

We next sequenced samples isolated from different zones of histologic progression within the same AIS and MIA tumours (five patients each), as described in Fig. 1. Three or four histologically different zones were collected from each AIS and MIA samples, respectively. The average high-quality coverage depths of tumour and normal DNA were 258 × and 349 × for AIS (Supplementary Table 7) and 450 × and 485 × for MIA (Supplementary Table 8). Using innovative pipeline-based analysis we identified 21 and 30 unique non-synonymous aberrations across the AIS and MIA patients, respectively (Supplementary Table 9), with an average mutation rate of 6.2 and 10.8 mutations per patient (Supplementary Table 10). Although sampling additional tumour regions (zone 4) may contribute to the overall higher mutations rate in MIA patients, the amount of mutated genes was equal in both cohorts (Supplementary Table 10), suggesting that several genes bearing multiple mutations occur in different MIA patients. Indeed, we found that *EGFR* and *TP53* were the most frequently mutated genes in MIA patients, followed by *CTNNB1*, *MED12* and *ATRX* (Supplementary Table 11). Four of five MIA patients harboured mutations in the EGFR tyrosine kinase domain and mutations in the *TP53* DNA-binding domain were found in three of five MIA cases (Table 2; Supplementary Table 12). Interestingly, two recurrent mutations (in two MIA patients each), were found in *EGFR* and *TP53* (Supplementary Table 13). Additionally, aberrations in the *NOTCH* family (in-frame deletion in *NOTCH1-*PEST domain, p.1700D>Y substitution in *NOTCH3* and amplification of *NOTCH4*) were detected in three MIA patients (predominantly in the invasive zone 4). Four MIA patients harboured mutations in the WNT pathway genes (*CTNNB1*, *APC* and *MED12*)^25^ (Table 2; Supplementary Table 12). Although WNT pathway mutations are uncommon in lung cancers they emerge in a subset of NSCLC adenocarcinomas^26^ and consecutive activation of WNT pathway by epigenetic silencing of WNT antagonists supports the crucial role of WNT signalling during progression towards malignancy^27^.

In contrast to MIA, the mutational landscape varied considerably across the lesions in five AIS patients. The only gene that was mutated in two different AIS lesions was *ARID1A*, a component of the SWI/SNF chromatin remodelling complex (Table 3; Supplementary Table 11). One lesion out of five harboured a mutated *TP53* or *EGFR* gene and one lesion had *NOTCH1* amplification (Table 3). Unlike in MIA, only one AIS lesion carried mutated *APC* gene, suggesting that disruption of WNT signalling plays less significant role in AIS carcinogenesis. Moreover, alterations in growth factor receptors other than EGFR were seen in four of five AIS tumours (Table 3). Six genes were mutated in both AIS and MIA patients (Supplementary Table 14), suggesting the potential role of these events in the transition from non-invasive to invasive disease. Eleven of 21 mutations found in AIS and 10 of 30 mutations identified in MIA have been previously reported in various malignancies (Supplementary Table 15). In both groups, we revealed mutations in genes that are rarely implicated in lung adenocarcinoma (Supplementary Table 16). Similar to AAHs, genes associated with DNA repair and chromatin remodelling network, ATM^20^, MSH6 (ref. 20), ARID1A/ARID1B^24^, PBRM1 (ref. 28), ASXL1 (ref. 21), ATRX^22^, BRCA2 (ref. 29) and CREBBP^30^, were mutated in most AIS and MIA tumours (Tables 2 and 3; Supplementary Table 12).

### Mutational landscape varies along tumour progression

**Figure 2:**
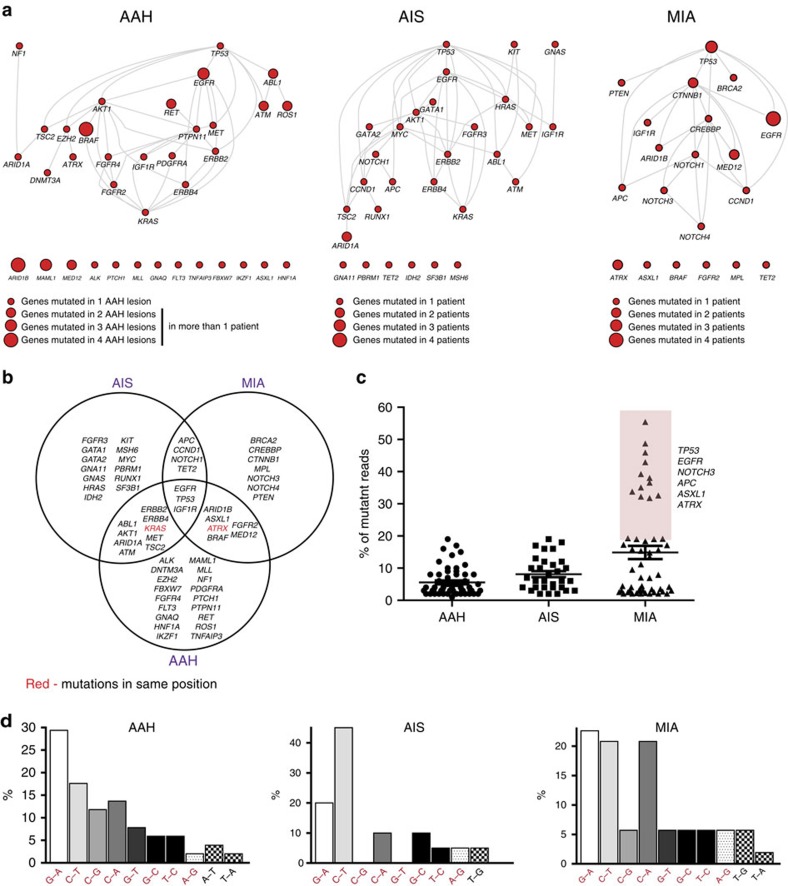
Mutational landscape varies between the three stages of tumour progression. (**a**) Cytoscape bioinformatics toolset was used to create the network of protein–protein interactions of the mutated genes in each respective progression stage. Red nodes indicate the mutated genes, with node size dependent on number of mutations found in AAH, AIS and MIA patients. (**b**) Venn diagram was created to compare genes mutated in different stages of lung adenocarcinoma evolution (in red—mutations in the same position). (**c**) The chart demonstrates the percentage of fractional abundance of all mutations found in AAH, AIS and MIA. Pink frame in MIA cohort indicates genes that harbour mutations with fractional abundance higher than 20%. (**d**) Spectrum of base-pair substitutions in AAH lesions and AIS or MIA tumours (in red, base-pair substitutions that present in all three groups). Silent mutations were not included in the analysis.

To better visualize the dynamics of genetic changes in glandular tumorigenesis, we used Cytoscape bioinformatic toolset to generate protein–protein interaction networks of the mutated genes in each respective progression stage (Fig. 2a). Red nodes indicate the mutated genes, with node size dependent on number of mutations found in AAH, AIS and MIA patients. While multiple AAHs or MIA tumours may share common alterations (such as BRAF or EGFR, respectively), specific mutations are limited to individual patients in relatively more heterogeneous AIS tumours (Fig. 2a; Supplementary Fig. 1). The observation that AAH has the most mutations in genes without annotated interactions (13 as compared with 6 in both AIS and MIA) (Fig. 2a) is suggestive of higher diversity in potentially affected molecular pathways in pre-neoplastic lesions. However robust association of clonal events with signalling pathways warrants future study with whole exome sequencing.

While there were substantial overlaps in the affected genes and subsequent molecular interactions between the each step of histologic progression, the majority of mutations were specific to AAHs, AISs or MIAs (Fig. 2b), with only *EGFR*, *IGF1R* and *TP53* mutated in all stages. Of these, both *EGFR* and *TP53* are highly connected nodes in the mutational landscape (Supplementary Fig. 1), consistent with their potential as drivers of glandular tumorigenesis. Similarly, mutation frequencies in *EGFR* and *TP53* sequentially increased from early lesions to MIA (Fig. 2a). Interestingly, a significant increase in mutation fractional abundance of these genes was also seen in MIA lesions, compared with AAH and AIS (Fig. 2c). We next analysed somatic substitutions in AAH, AIS and MIA. The most frequent base-pair substitution in AAH and MIA samples was G–A, followed by C–T, C–A and C–G (Fig. 2d), whereas in AIS, mutations were scattered with a higher C to T transition frequency and decreased C–A transversions, while C to G mutations were not present. Interestingly, an increase in C–T substitutions was associated with a higher mutation rate and broad mutational spectrum in lung tumours^3^. This observation is consistent with the more heterogeneous mutational landscape of AIS relative to AAH or MIA.

### Analysis of AAHs and matched primary adenocarcinoma

A single biopsy of primary invasive adenocarcinoma was available for each one of the six patients whose lungs were used for collection of AAHs. DNA isolated from these tumours was sequenced and the mutational spectrum was compared with the multiple AAHs collected from the same lung. Only one of six primary tumours harboured *BRAF* mutation. Moreover, three of four patients with mutated *BRAF* in AAHs carried wild-type *BRAF* in paired invasive adenocarcinoma (Table 1). In two patients, we identified three possible cancer-driving mutations (*EGFR* deletion, TP53 p.279G>E and KRAS p.12G>C substitutions) in several AAHs and matched primary tumours samples (Table 1). Interestingly, in all three cases we saw a consecutive increase in mutations fractional abundance from AAH to primary tumour (Table 4), suggesting a spatial expansion of these clones as they progressed histologically.

### Harbingers of malignant transition

Our analysis revealed that two of three *TP53*-mutated MIAs (Table 2) and two *TP53*-mutated AAH lesions (Table 1) also carried activating *EGFR* mutations. Moreover, an activating mutation in *EGFR* and concurrent loss-of-function mutation in *TP53* were seen in two distinct AAHs and primary invasive adenocarcinoma derived from the same patient (Table 1). These data imply that concomitant TP53 silencing and EGFR activation may favour malignant transformation. Likewise, a *KRAS* p.12G>C substitution was spotted in three AAH lesions and primary adenocarcinoma within the same lung (Tables 1 and 4), indicating that *KRAS* mutation is also an early transformation-driving event in multistep progression.

### Intratumour heterogeneity in early lung neoplasms

**Figure 3:**
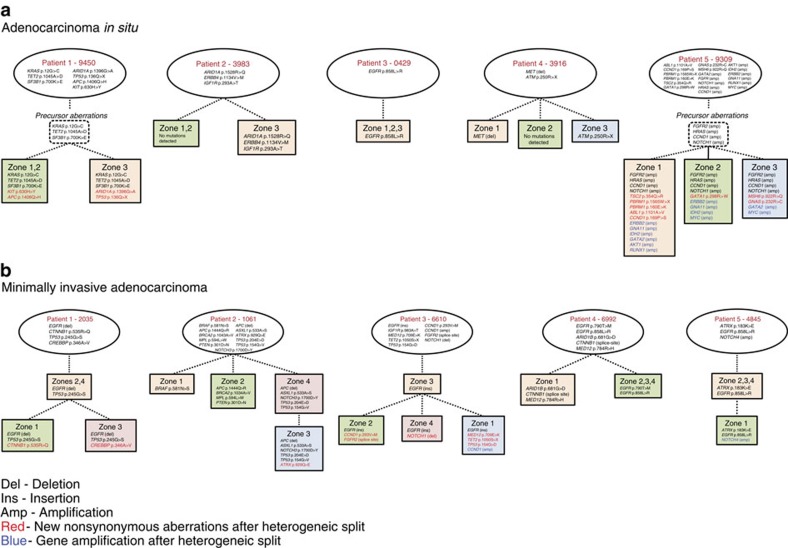
Intratumour heterogeneity and branched evolution in AIS and MIA tumours. Dendrograms relating relative development of mutations from inferred common ancestors (dashed, white rounded boxes) and tumour zones (solid boxes) for each (**a**) AIS and (**b**) MIA tumour. A complete list of mutations in each patient is provided in the white ovals. Mutations in common ancestors are shown in black, while subsequent alterations are depicted in red for mutations and blue for copy number variations. Zones with no mutation (zones 1 and 2 for AIS patient #2 and zone 2 for AIS patient #4) are shown as parallel branches, but may also be ancestors of subsequent mutations in other zones. Synonymous and non-coding mutations were not considered in the analyses.

Accumulating evidence suggests that tumours often evolve through a process of branched evolution, involving genetically distinct subclones^31^, although the timing of this phenomenon during the early stages of tumour evolution is lacking. Sequencing of samples collected from different zones within the same AIS or MIA tumours revealed a large degree of heterogeneity among different foci (Fig. 3a,b). While some mutations were seen in all foci collected from different zones, others were restricted to only one focus. Mutations present in all zones within the same lesion were limited to a handful of well-known cancer-driving genes, such as *EGFR*, *TP53*, *KRAS* and *ATRX* (Tables 2 and 3 and Fig. 3), supporting previous evidence that mutations in known driver genes occur early in cancer evolution 32. Nevertheless, 81% (AIS) and 83% (MIA) of all somatic mutations were not detectable across every focus of a given lesion. Although clonal evolution with distinct mutations in different zones was already present in AIS (Fig. 3a), the branched evolution of MIA is consistent with subsequent expansion of these clones, with common mutations observed across several zones (Fig. 3b).

### Detection of lesion-associated DNA in bodily fluids

**Figure 4:**
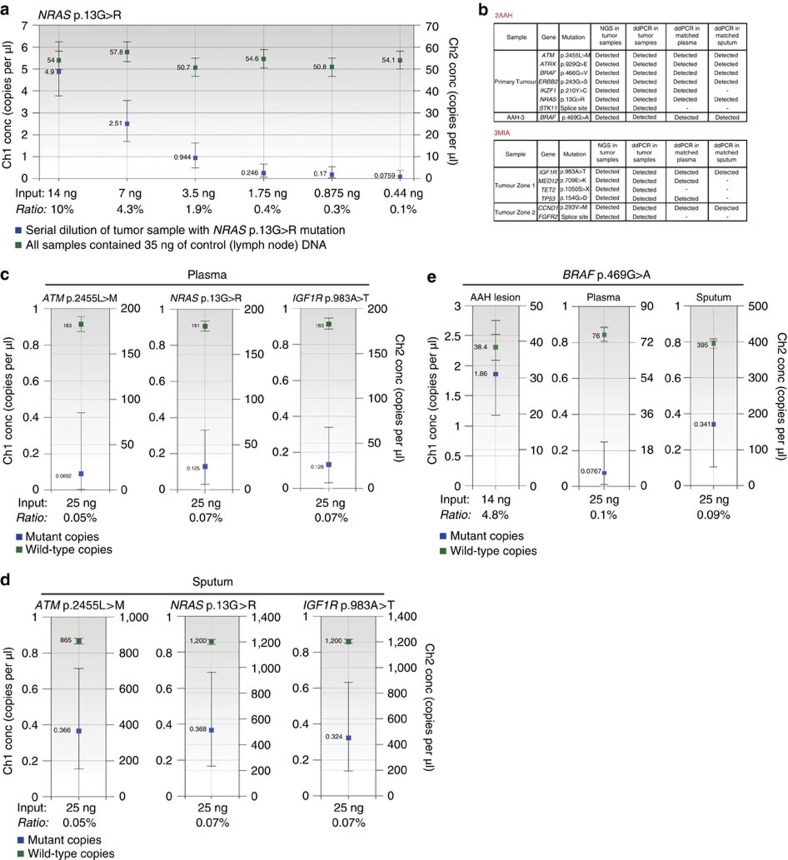
Detection of lesion-associated DNA in bodily fluids. (**a**) DNA with known *NRAS* mutation (extracted from the primary tumour of patient 2AAH) was serially diluted with 35 ng of the matched control DNA isolated from the lymph node of the same patient and every mixed mutant:wild-type sample was assessed using PrimePCR ddPCR assay. The blue markers indicate the concentration of mutant DNA (copies per μl) and the green markers indicate the concentration of wild-type DNA (copies per μl) in each sample. All error bars generated by QuantaSoft software represent the 95% confidence interval. Fractional abundance of the mutant DNA in a wild-type DNA background is shown at the bottom of the plot. (**b**) Table shows the list of mutations detected either in primary lesions, blood or sputum samples from two patients, analysed using NGS or *PrimePCR ddPCR* assay. (**c**) Mutant circDNA has been detected by *PrimePCR ddPCR* assay in blood and (**d**) sputum samples isolated from patients with known *ATM*, *NRAS* and *IGF1R* mutations. Fractional abundance of these mutations in primary tumours was 35%, 20% and 2% respectively. (**e**) *BRAF* mutation that was identified in pre-malignant AAH lesion was detected in DNA extracted from matched plasma and sputum species by ddPCR.

We used ddPCR to analyze DNA samples extracted from plasma (collected at the time of surgery) or sputum of two patients (patient #3 from the MIA cohort (3MIA) and patient #2 from the AAH cohort (2AAH)) for the presence of mutations identified in their primary tumours by NGS. To evaluate assay sensitivity we serially diluted DNA with known *NRAS* p.13G>R mutation with normal DNA isolated from the lymph node of the same patient. The fractional abundance plot (Fig. 4a) shows that even 0.1% of the mutant DNA can be detected in a wild-type background. We next validated each ddPCR assay for mutation detection in the DNA samples that were used for NGS. All 14 mutations identified by NGS were also detected by ddPCR (Fig. 4b) and the prevalence of the mutant reads were very consistent between the NGS and ddRPC assays. We next analysed DNA samples extracted from plasma or sputum of both patients for the presence of 13 mutations identified in their primary tumours. Of 13 mutations tested, 10 were detected in plasma and 7 were detected in the sputum samples (Fig. 4b). We detected mutated *IGF1R*, *MED12*, *TP53* and *CCND1* genes in plasma DNA of the 3MIA patient even though all these mutations were identified by deep sequencing in only one of four zones selectively sampled from the primary tumour of this patient (Table 2). Interestingly, there was no correlation in mutant to wild-type fractional abundance between the primary tumour and bodily fluids. For instance, while fractional abundance of *ATM*, *NRAS* and *IGF1R* mutations in the primary tumour was 35%, 20% and 2%, respectively, the prevalence of these mutations in the corresponding plasma samples was 0.05, 0.07 and 0.07% (Fig. 4c), whereas in the sputum, mutations fractions were 0.04, 0.03 and 0.03% (Fig. 4d). Surprisingly, in DNA isolated from plasma and sputum of an AAH patient, we were able to detect a low prevalence *BRAF* p.469G>A mutation that was identified by NGS in DNA extracted from the AAH lesion of this patient (Fig. 4e). This mutation was not detected in the matched primary invasive tumour using ddPCR.

## Discussion

We sequenced recognized cancer-driving genes to uncover the timing and sequence of specific genetic alterations along the histologic continuum preceding overt pulmonary malignancy. Analysis of multiregion sequencing within individual AISs and MIAs revealed evidence of intratumour heterogeneity even in early non-invasive AIS tumours. Although the spatial intratumour heterogeneity in overt NSCLC have bees recently reported^33^, our work further supports the theory of branched evolutionary tumour growth^33^,^34^ and for the first time, demonstrates heterogeneity of the clonal events in pre-invasive and minimally invasive lesions.

Neoplasms are thought to progress to cancer in part through genetic instability. Interestingly, we identified mutations in genes associated with DNA repair in most early lesions. Since DNA repair plays a pivotal role in maintaining genomic integrity^35^, dysregulation of DNA repair machinery may be a driving force behind the early onset of spatial heterogeneity in pre-malignant lesions and may further contribute to tumour oligoclonality. Consistent with this data, mutations associated with good and poor prognosis or responses to therapy were seen in different regions of the same tumour. For example, *TP53* alterations, which are typically associated with poor prognosis in patients with pulmonary adenocarcinoma^36^, were seen in some, but not all, zones of one AIS patient and one MIA patient. The *EGFR* p.858L>R mutation, which renders cells sensitive to the EGFR inhibitor gefitinib^37^,^38^, was found in zones 2–4 of one MIA patient, but was absent from zone 1. It is therefore important to understand the limitations of data derived from single biopsies that are often used to predict therapeutic responses in the clinic.

Current models of tumorigenesis hold that lung adenocarcinomas do not arise spontaneously, but begin as microscopic clonal cellular proliferations that are driven through progressive histologic stages by the acquisition of genetic and epigenetic alterations. Our data reveal that the majority of discovered mutations and potentially affected molecular pathways are specific to histologically distinct subtypes of these defined lesions. In fact, among the 125 cancer genes sequenced, only *EGFR*, *TP53* and *IGF1R* were mutated across the full histologic spectrum of early glandular neoplasia. In addition, genes affected in a specific pathway vary from tumour to tumour and only a fraction of tumours gain aberrations in well-established cancer-driving genes (for example, *TP53*, *EGFR* or *RAS*), suggesting that there is no single predominant pattern of lung cancer progression. Although these findings are likely biased by the panel of highly selected genes, this panel revealed important differences in potential drivers along lung cancerogenesis. Taken together, our results indicate that lung adenocarcinoma may arise from different molecular pathways and support the concept that some mutations are indeed dead ends and do not progress to more invasive cancers, unless they represent the necessary predisposition for truly precursor of invasive adenocarcinoma^32^,^39^. We acknowledge that due to limited samples size, our findings are exciting but exploratory, and are therefore restricted to hypothesis generation. However, given the difficulty of obtaining such specimens, our samples collection represents a unique model to study genetic alterations that initiate and drive lung cancer progression. Nevertheless, larger confirmatory studies using longitudinal samples (that allow tracking a clonal population over time) and precise calculation of the percentage of abnormal cells containing each variant are warranted to further validate our observations and provide further insight into fundamental biologic mechanisms of evolutionary tumour growth.

*BRAF* was the most frequently mutated gene in AAH lesions (four patients carry two recurrent 469G>A substitutions and two mutations at positions 601K and 594D), yet three of four patients with mutated *BRAF* in AAHs carried wild-type *BRAF* in the matched invasive adenocarcinoma and no *BRAF* mutations were identified in the invasive core (zone 4) in any of five MIA patients. Consistently, the frequency of *BRAF* somatic mutations in overt adenocarcinoma is low (about 2%)^40^. Therefore, it is most likely that the invasive clone rarely develops from a lesion with a *BRAF* mutation. On the other hand, the p.469G>A and p.601K>E mutations found in AAH samples are activating, and enhance the ability of BRAF to phosphorylate and activate MEK^41^,^42^, while deactivating D594 mutations result in the hyperactivation of MEK signalling in the presence of activated KRAS^43^. Taken together, our findings indicate that *BRAF* mutations are likely to stimulate proliferation and induce hyperplasia, however early lesions with mutated *BRAF* rarely progress to malignant disease, unless they are overlapping with other oncogenic mutations found in NSCLC.

*EGFR* and *TP53*—well known for their role in malignant lung carcinomas^12^,^44^, appear to represent important targets in the earliest stages of lung tumour development. Not only were these gene mutations present in each of the early lesions, but the frequency of *EGFR* and *TP53* mutations increased with each advancing step of histologic progression and were almost always present by the time a lesion had reached the MIA stage. This observation supports the spatial expansion of critical genetic events during early tumorigenesis, a view that is further supported by the observation that MIAs harbour a considerably higher fractional abundance of *EGFR* and *TP53* mutations than AAHs and AISs. The view that *EGFR* and *TP53* mutations are initial events that drive early tumour progression is supported by several studies that demonstrate sequential increase in *EGFR* alterations^9^ and intra-nuclear accumulation of nonfunctional P53 protein throughout the AAH–AIS–MIA continuum^45^,^46^. Moreover, transgenic mouse models expressing mutated *EGFR* in type II pneumocytes develop AAH, BAC and invasive adenocarcinoma in a time-dependent manner^47^. The types of mutations involving *EGFR* and *TP53* in these early glandular neoplasms are not inconsequential to tumour development. All of the *EGFR* mutations were activating mutations within the *EGFR* tyrosine kinase domain that have been previously reported in non-small cell lung carcinomas in association with clinical response to EGFR tyrosine kinase inhibitors^48^,^49^. The *TP53* mutations were contained within the DNA-binding domain, the site typically associated with P53 inactivation^50^. This association suggests that *TP53* silencing and *EGFR* activation favors malignant progression of early glandular neoplasms. Of note, concurrent *TP53* and *EGFR* mutations were detected in two of three *TP53*-mutated MIAs and in two AAHs and the associated invasive adenocarcinoma collected from the same lung. These findings are consistent with previous studies, which demonstrated that persistent EGFR signalling from activating mutations is necessary for the development and maintenance of lung adenocarcinomas^51^, and functional P53 inactivation may facilitate the transition from non-invasive to invasive tumour growth^12^,^45^,^46^. Furthermore, a recent observational study by McGranahan *et al.*^32^ using publicly available TCGA database, indicated that most of the *EGFR* and *TP53* somatic mutations in lung cancer are clonal and typically occur early in tumour evolution, likely even before tumorigenesis. Therefore, our findings further support the notion that coinciding loss of P53 activity and EGFR activation may signal the transition to invasive tumour growth, and that EGFR mutation may actually better define specific subtypes of early lesions less likely to progress to overt invasive carcinoma.

In addition to *EGFR* and *TP53* aberrations, *KRAS* mutation was also seen in AAHs and paired adenocarcinoma. Consistent with other studies^8^,^32^, these findings indicate that *KRAS* mutation is also an early event in the multistep lung carcinogenesis. While the genetic relationship between AAHs and the associated adenocarcinoma is not yet defined, it is possible that these mutations result due to a multifocality of the carcinogenetic process^52^, suggesting a field cancerisation state in lung cancer development^53^, since *KRAS* mutation is specifically linked to smoking exposure. Alternatively, given the fact that all three mutations are well known cancer-drivers in various solid tumours including lung^54^,^55^,^56^,^57^, and that in all cases we saw a consecutive increase in mutations fractional abundance from AAH to primary tumour, it is possible that these genomic alterations underwent distant spatial expansion along the lung epithelium. In this case, our results may support a clonal relationship between adenocarcinoma and AAHs present simultaneously in the same lung. Nevertheless, further studies with larger cohorts will be needed to define whether in some cases, AAHs and paired adenocarcinoma are clonally related^52^ to each other or represent independent neoplastic foci.

Since it is difficult to obtain multiple lung tumour biopsies to assess tumour heterogeneity we used ultra-sensitive ddPCR to analyze DNA samples extracted from plasma or sputum of two patients (one MIA case and one invasive adenocarcinoma patient with multiple AAH lesions) for the presence of mutations identified in their primary tumours by NGS. The majority of the mutations were readily detected in bodily fluids. The lower detection rate in sputum may be attributed to the anatomical situation of the neoplasm^58^, presence of high-DNase activity^59^ and subsequent DNA degradation^60^. Surprisingly, in plasma we detected mutations that were identified by NGS in only one of four zones selectively sampled from the primary tumour, indicating that circDNA is highly representative of the intratumour mutational heterogeneity. Interestingly, our results indicate lack of correlation in mutant to wild-type fractional abundance between the primary tumours and matched bodily fluids. This phenomenon has been previously seen in early stage breast cancer patients^61^ and a possible explanation may come from changes in vascularization and cell death rate across different tumour areas. In free circDNA of one patient we detected a low prevalence of the *BRAF* p.469G>A mutation that was identified by NGS and ddPCR in DNA extracted from the AAH foci but was not found in the overt adenocarcinoma collected from the same lung. Although a very rare presence of this mutation in the primary invasive tumour cannot be completely excluded and it does not necessarily support the malignant potential of this AAH lesion, our data provide proof of concept that genetic alterations associated with very small early glandular neoplasms can be detected in paired circulating DNA even before they invade and acquire malignant potential.

Collectively, our study provides the unique insight into the genetic alterations that initiate and drive the progression of lung glandular neoplasia and underlines the need for precise definition of these events to improve proper diagnosis and early detection of tumours. Identification of mutational features which characterize relevant lesions that actually progress to cancers will allow to better predict the fate of these early lesions and tailor the right therapy to prevent the progression.

## Methods

### Samples

All samples were obtained following IRB-approved protocols. Approval for research on human subjects was obtained from the Johns Hopkins University institutional review boards. This study qualified for exemption under the U.S. Department of Health and Human Services policy for protection of human subjects [45 CFR 46.101(b)] (IRB study number—00076266). Formalin-fixed, paraffin-embedded lung cancer specimens harbouring multiple AAH lesions and AIS or MIA tumours were retrospectively collected from the Johns Hopkins Medical Institutions tissue archive. Patient’s characteristics including gender, race, age and smoking history are summarized in Supplementary Table 17 and clinical diagnosis is summarized in Supplementary Tables 18–20. DNA was extracted from 25 distinct AAHs incidentally discovered in the lung resection specimens from six patients with invasive adenocarcinoma. AAH is the earliest form of glandular neoplasia of the lung, which characterized by the proliferation of slightly atypical epithelial cells lining the slightly thickened by intact septae. Samples from AIS and MIA tumours extracted from five patients each were collected from different zones of histologic progression within the same lesion. Three or four histologically different zones were collected from each AIS and MIA samples respectively. AIS is characterized by atypical cells with enlarged hyperchromatic nuclei lining intact alveolated lung parenchyma (zone 1). With progression towards the centre of the tumour, the septae become increasingly thickened and the cytologic atypia becomes more pronounced (zone 2). The central area of greatest cytoarchitectural atypia was selectively sampled in the AISs as (zone 3). In the MIAs, an additional zone (zone 4) was included to capture the small focus showing invasive tumour growth (<0.5 cm).

### Samples validation and DNA extraction

All samples were reviewed by a senior pathologist, Dr William Westra, to reconfirm the diagnosis and were classified according to recent classification schemes for early glandular neoplasms of the lung^4^. Paraffin-embedded slides were microdissected to obtain >60% neoplastic cells. Neoplastic cellularity was estimated from the sequential slides, which highly reflect cellularity of the section used for DNA sequencing. DNA was extracted using standard protocols as previously described^62^ and quantified with Nanodrop system (Thermo Scientific). As a control, matched normal lymph nodes free of atypical cells were used in each case. At least 250 ng DNA has been used for the library construction and subsequent targeted sequencing. Plasma and sputum was collected from two individuals with histologically confirmed cases of primary lung cancer. Plasma and sputum DNA was extracted by digestion with 50 μg ml^−1^ proteinase K (Boehringer Mannheim) in the presence of 1% SDS at 48 °C overnight followed by phenol/chloroform extraction and ethanol precipitation^63^.

### Analytical approach

Our goal was to implement the first inventory of the most common somatic mutations during the early steps of lung adenocarcinoma pathogenesis. To this end we have analysed all specimens available from our pathological archive for which multiple regions and histological subtypes could by identified and for which viable DNA could be obtained (see Sample section above). Further, we have implemented an analytic pipeline to identify, classify, and report all somatic mutations reliably identifiable across multiple specimens from the spatially and histologically defined lesions obtained from each patient. Due to the overall total limited number of individuals in each group available for the study comparative analyses were of limited value, we therefore focused on descriptive summaries of the findings. Hence, we have reported the total number of unique somatic mutations, the type of specific mutations and the mutation spectra within and across individuals, and along the outlined progression groups. We have further identified the most common mutations across all progression groups.

### *CancerSelect-R* panel

The *CancerSelect-R* panel analyzes the regions of 125 well-characterized cancer genes to identify tumour-specific (somatic) mutations, copy number changes and translocations. This panel contains drivers common to many solid tumour types, including genes that are clinically actionable in NSCLC. The genes were selected based on targets of FDA approved therapies, genes which when mutated would allow for enrolment onto a clinical trial, genes which have been demonstrated to provide clinical utility in published prospective or retrospective clinical trials, along with genes which are the targets of therapies in late-stage clinical development. The *CancerSelect* assay has been CLIA (Clinical Laboratory Improvement Amendments) validated and is reported as having a sensitivity of >99% and a specificity of >99.99% for the detection of mutations using a 2% cutoff.

### Sequencing

Sample library construction, targeted capture and NGS of tumour and normal samples were performed at PGDx (Baltimore, MD, USA). In brief, genomic DNA from tumour and normal samples were fragmented and targeted regions were captured in solution using the custom designed probes for 125 genes according to the manufacturer’s instructions (Agilent, Santa Clara, CA, USA). Paired-end sequencing, resulting in 150 bases from each end of the fragments, was performed using a MiSeq System (Illumina, San Diego, CA,USA). Raw NGS data has been uploaded to the NCBI Sequence Read Archive repository. Accession code for deposited data is PRJNA281261.

### Data analysis

Bioinformatic analyses of tumour and normal samples were performed at PGDx (Baltimore, MD, USA). Somatic mutations were identified using VariantDx custom software for identifying mutations in matched tumour and normal samples^64^,^65^. Prior to mutation calling, primary processing of sequence data for both tumour and normal samples were performed using CASAVA (v1.8) software (Illumina, San Diego, CA,USA), including masking of adapter sequences. Sequence reads were aligned against the human reference genome (version hg18) using ELAND algorithm of CASAVA software with additional realignment of select regions using the Needleman–Wunsch method. Candidate somatic mutations, consisting of point mutations, insertions and deletions were then identified using VariantDx across the regions of interest. In brief, an alignment filter was applied to exclude quality failed reads, unpaired reads, and poorly mapped reads in the tumour. A base quality filter was applied to limit inclusion of bases with reported phred quality score >30 for the tumour and >20 for the normal. A mutation in the tumour was identified as a candidate somatic mutation only when (i) distinct paired reads contained the mutation in the tumour; (ii) the number of distinct paired reads containing a particular mutation in the tumour was at least 2% of the total distinct read pairs and (iii) the mismatched base was not present in >1% of the reads in the matched normal sample as well as not present in a custom database of common germline variants derived from dbSNP and (iv) the position was covered in both the tumour and normal. Mutations arising from misplaced genome alignments, including paralogous sequences, were identified and excluded by searching the reference genome. Candidate somatic mutations were further filtered based on gene annotation to identify those occurring in protein coding regions. Functional consequences were predicted using snpEff and a custom database of CCDS, RefSeq and Ensembl annotations using the latest transcript versions available on hg18 from UCSC ( https://genome.ucsc.edu/). Predictions were ordered to prefer transcripts with canonical start and stop codons and CCDS or Refseq transcripts over Ensembl when available. Finally mutations were filtered to exclude intronic and silent changes, while retaining mutations resulting in missense mutations, nonsense mutations, frameshifts or splice site alterations. A manual visual inspection step was used to further remove artifactual changes. Copy number alterations were identified by comparing normalized average per-base coverage for a particular gene in a tumour sample to the normalized average per-base coverage in a matched normal sample for the patient (Supplementary Tables 21–23).

### Digital PCR

All ddPCR assays used in this study were designed and optimized to work in the ddPCR system by Bio-Rad (Hercules, CA, USA). The Bio-Rad assays IDs are summarized in Supplementary Table 24. The ddPCR mixture contained 8 μl of 2 × ddPCR Supermix (Bio-Rad), 400 nM of forward and reverse primers, 125 nM mutant and wild-tube probe and 2 μl DNA isolated from primary tumour, AAH lesion, plasma or sputum in each 20 μl reaction. The entire 20 μl reaction was loaded into a droplet cartridge (Bio-Rad), a gasket placed over the cartridge according to the Bio-Rad protocol and the cartridge placed in the droplet generator (Bio-Rad #186-3002). Once inside the droplet generator a vacuum was applied to the cartridge. This draws both the PCR reagents and oil through a flow-focusing nozzle where around 20,000 individual droplets ∼1 nl in size are formed, suspended in an emulsion. The emulsion was transferred into a 96 well plate (Eppendorf, Hamburg, Germany) and sealed using a foil lid and a thermal plate sealer (Bio-Rad). Sealed plates were cycled using a C-1000 thermal cycler (Bio-Rad) under the following conditions: 10 min hold at 95 °C, 45 cycles of 95 °C for 15 s then 60 °C for 60 s. After amplification, the plate was transferred to a Bio-Rad droplet reader from which raw fluorescence amplitude data was extracted from the Quantasoft software for downstream analysis.

### Phylogenetic analysis

Branched evolution of tumours within each patient was inferred by comparing lists of mutations in each tumour zone. A zone containing all mutations observed in another zone was indicated as its ancestor. If no such zone existed, putative precursors were inferred from the set of alterations common to multiple zones. Zones with no alterations were considered parallel branches, although an alternative dendrogram may be formed by assuming that these zones are ancestors of zones with mutations.

### Network analysis

Mutation networks were generated with Cytoscape (v3.1.0) (ref. 66). The GeneMANIA plugin^67^ was used to merge interactions in version 2013-10-15 between query genes annotated in Pathway Commons^68^, Wu *et al.*^69^, and the Human Protein Reference Database^70^. Queries were conducted on all genes tested in the panel or the subset of mutated genes in each lung cancer stage. No related genes to query genes were included in the search.

## Additional information

**Accession codes:** The next-generation sequencing data have been deposited in the NCBI Sequence Read Archive repository under accession code PRJNA281261.

**How to cite this article:** Izumchenko, E. *et al.* Targeted sequencing reveals clonal genetic changes in the progression of early lung neoplasms and paired circulating DNA. *Nat. Commun.* 6:8258 doi: 10.1038/ncomms9258 (2015).

## Supplementary Material

Supplementary Information Supplementary Figure 1 and Supplementary Tables 1-24

## Figures and Tables

**Table 1:**
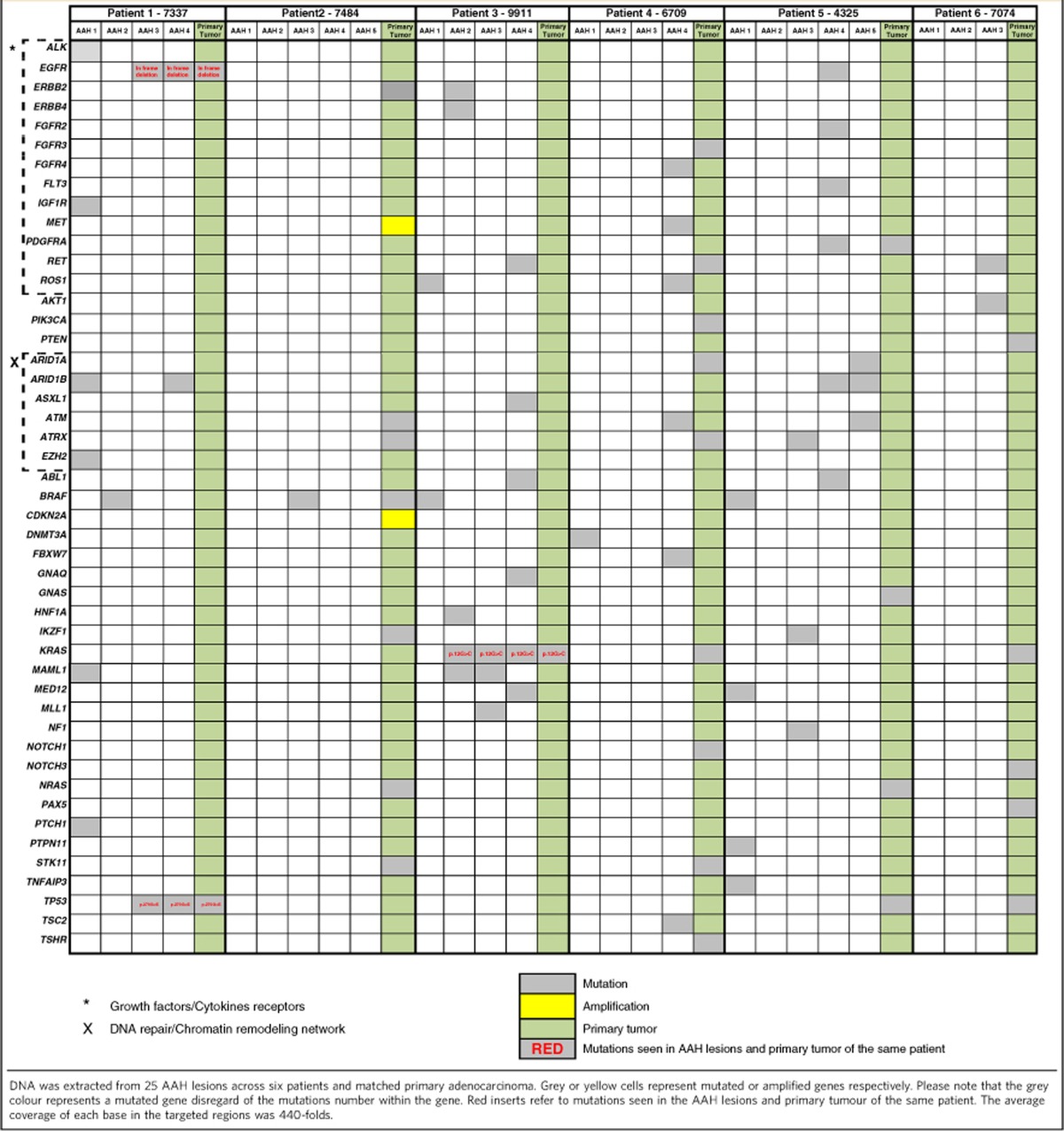
Mutations landscape in pre-malignant AAH lesions.

**Table 2:**
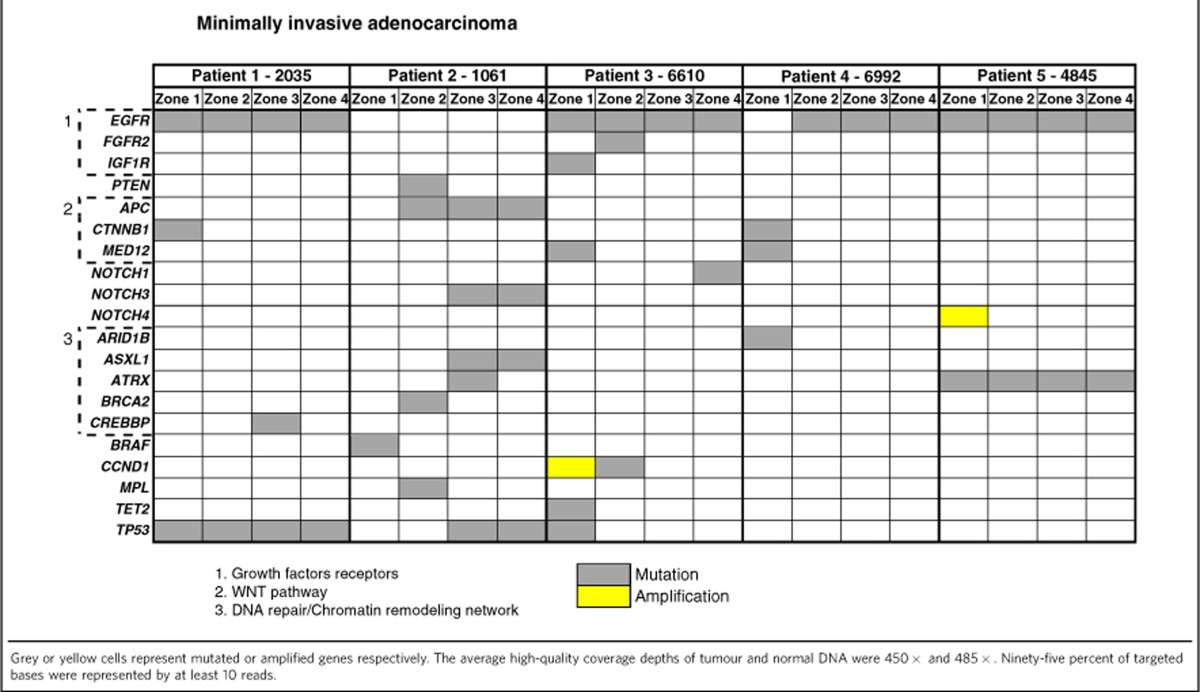
Mutations landscape in MIA tumours.

**Table 3:**
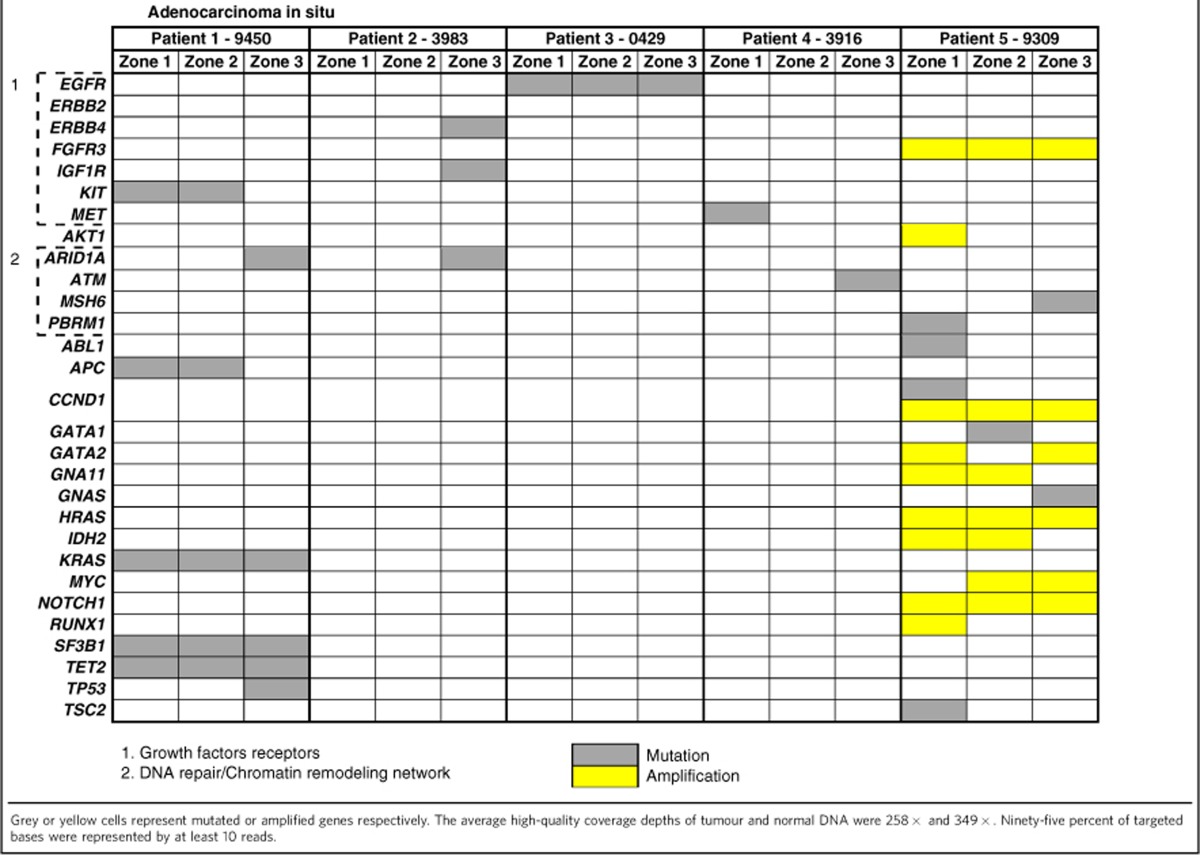
Mutations landscape in AIS tumours.

**Table 4:** Fractional abundance of mutations in AAHs and matched adenocarcinoma.

	**Patient 1—7337**				**Patient 3—9911**			
	**AAH3 (%)**	**AAH4 (%)**	**Primarytumour (%)**		**AAH2 (%)**	**AAH3 (%)**	**AAH4 (%)**	**Primarytumour (%)**
*EGFR*—in-frame del	2	2	9	*KRAS*p.12G>C	6	8	8	13
*TP53* p.279G>E	3	17	20					

The fractional abundance of indicated mutations in pre-neoplastic lesions and matched primary adenocarcinoma resected from the same lung.
