# Supplementary material for: Targeted sequencing reveals clonal genetic changes in the progression of early lung neoplasms and paired circulating DNA
Source: Nat Commun. 2015 Sep 16;6:8258. doi: 10.1038/ncomms9258 (PMC4595648; doi:10.1038/ncomms9258)
Supplement: Supplementary Information — Supplementary Figure 1 and Supplementary Tables 1-24 [file ncomms9258-s1.pdf]

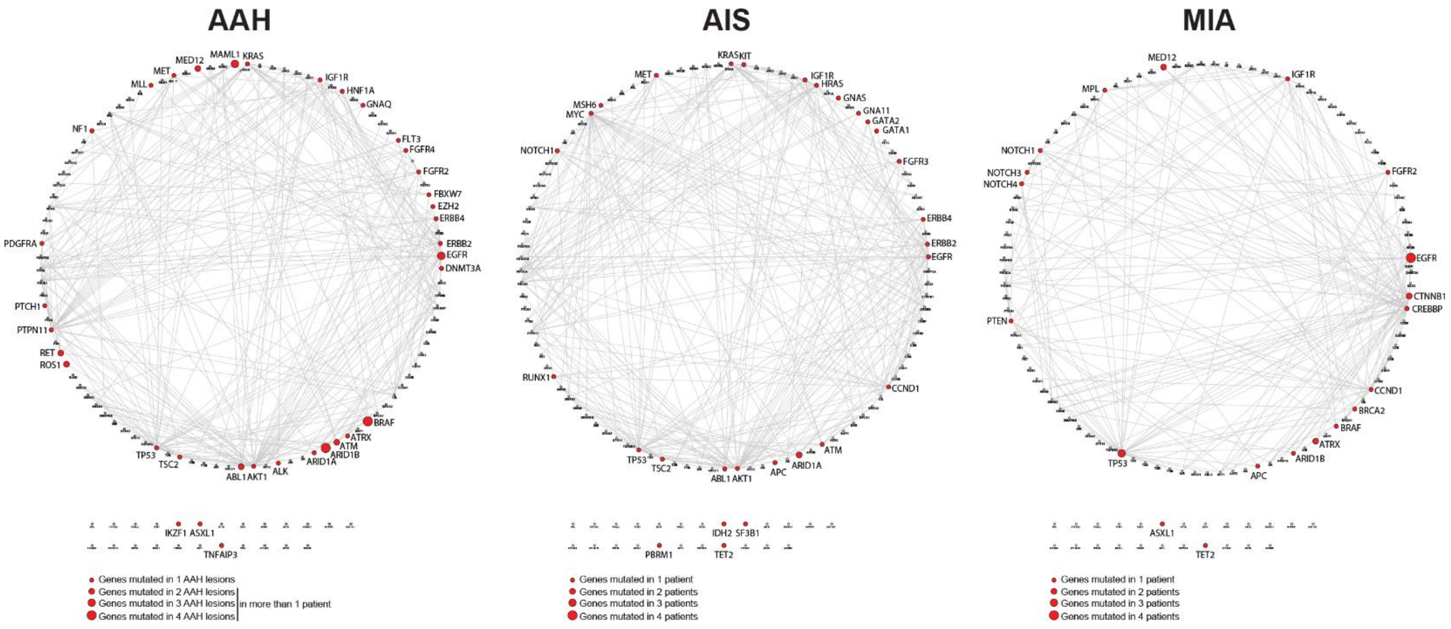

Supplementary Figure 1. Cytoscape bioinformatics toolset was used to create the network of protein-protein interactions between the product of each mutated gene and the panel of 125 cancer-driving genes profiled in this study. Red nodes indicate the mutated genes, with node size dependent upon number of mutations found in AAH, AIS and MIA patients.

Targeted Cancer Gene Analysis

Sequence and copy number analysis for coding regions of 125 well-characterized cancer and pharmacogenomic genes

ABL1	CYP1A2	GNAS	MTHFR	RNF43
AKT1	CYP2C19	HNF1A	MYC	ROS1
AKT2	CYP2C9	HRAS	MYCN	RUNX1
ALK	CYP2D6	IDH1	MYD88	SF3B1
APC	DAXX	IDH2	NF1	SMAD2
AR	DNMT3A	IGF1R	NF2	SMAD3
ARID1A	DPYD	IGF2R	NOTCH1	SMAD4
ARID1B	EGFR	IKZF1	NOTCH2	SMARCB1
ASXL1	ERBB2	JAK1	NOTCH3	SMO
ATM	ERBB3	JAK2	NOTCH4	STAG2
ATRX	ERBB4	JAK3	NPM1	STK11
BAP1	ERCC1	KDR	NRAS	TET2
BRAF	EZH2	KIT	PALB2	TGFBR2
BRCA1	FBXW7	KRAS	PAX5	TNFAIP3
BRCA2	FGFR1	MAML1	PBRM1	TP53
CBL	FGFR2	MDM2	PDGFRA	TPMT
CCND1	FGFR3	MDM4	PDGFRB	TSC1
CCNE1	FGFR4	MED12	PIK3CA	TSC2
CDH1	FLT3	MEN1	PIK3R1	TSHR
CDK4	FOXL2	MET	PMS2	TYMS
CDK6	G6PD	MLH1	PTCH1	UGT1A1
CDKN2A	GATA1	MLL	PTEN	VHL
CEBPA	GATA2	MPL	PTPN11	VKORC1
CREBBP	GNA11	MSH2	RB1	WT1
CTNNB1	GNAQ	MSH6	RET	XRCC1

Rearrangement analysis for selected introns of 17 well-characterized cancere genes

ALK	ETV1	EWSR1	RAF1	TMPRSS2
BCR	ETV4	MLL	RARA	
BRAF	ETV5	PDGFRA	RET	
EGFR	ETV6	PDGFRB	ROS1	

Supplementary Table 1. A *CancerSelect* panel for the detection of genetic alterations in 134 well-characterized cancer and pharmacogenomics genes used in this study. Design of the panel incorporates genes of high clinical and biologic importance that are screened using next-generation sequencing at extremely high coverage.

Atypical Adenomatous Hyperplasia

Patient # 7337	Normal control	Primary Tumor	AAH-1	AAH-2	AAH-3	AAH-4
Bases in Target Region	603600	603600	603600	603600	603600	603600
Read Length	150	150	150	150	150	150
Bases Sequenced (Filtered)	960023400	1159539900	822885300	1305471600	1050767100	1388796600
Bases Mapped to Genome (filtered)	511662600	606058500	521526300	770902050	548865300	1086099000
Percent Mapped to Genome	53%	52%	63%	59%	52%	78%
Bases Mapped to ROI	271212622	342297432	275712281	369929281	292263025	425442135
Percent Mapped to ROI	53%	56%	53%	48%	53%	39%
Targeted bases with at least 10 reads	577445	580125	579398	582830	578325	589218
Targeted bases with at least 10 reads (%)	96%	96%	96%	97%	96%	98%
Targeted bases with at least 10 distinct reads	573212	578693	577767	582482	576025	589131
Targeted bases with at least 10 distinct reads (%)	95%	96%	96%	97%	95%	98%
Average Raw Coverage	449	566	456	611	484	700
Average High Quality Coverage	441	554	448	600	474	684
Effective Coverage	0.98	0.98	0.98	0.98	0.98	0.98
SNPs in Tumor (> 0.4, Cov > 20 in both)	NA	107	104	102	104	131
Present in Normal	NA	107	104	102	104	131
Percent Present in Normal	NA	100%	100%	100%	100%	100%

Patient # 7484	Normal control	Primary Tumor	AAH-1	AAH-2	AAH-3	AAH-4	AAH-5
Bases in Target Region	603600	603600	603600	603600	603600	603600.00	603600
Read Length	150	150	150	150	150	150.00	150
Bases Sequenced (Filtered)	792603300	824583600	752885400	1127735100	1059993600	1056920400.00	1161861600
Bases Mapped to Genome (filtered)	299846550	424737300	377686200	745119300	620253450	433523550.00	449729400
Percent Mapped to Genome	38%	52%	50%	66%	59%	41%	39%
Bases Mapped to ROI	161493560	230286660	170911615	306366352	280518016	224836878.00	124281827
Percent Mapped to ROI	54%	54%	45%	41%	45%	52%	28%
Targeted bases with at least 10 reads	574481	576317	572362	581511	579477	576591.00	572074
Targeted bases with at least 10 reads (%)	95%	95%	95%	96%	96%	96%	95%
Targeted bases with at least 10 distinct reads	572257	575364	567427	581331	578991	575677	571305
Targeted bases with at least 10 distinct reads (%)	95%	95%	94%	96%	96%	95%	95%
Average Raw Coverage	268	382	284	506	464	371	206
Average High Quality Coverage	261	374	276	495	453	362	201
Effective Coverage	0.96	0.96	0.96	0.97	0.97	0.98	0.96
SNPs in Tumor (> 0.4, Cov > 20 in both)	NA	122	106	107	109	119.00	107
Present in Normal	NA	122	106	107	109	119.00	107
Percent Present in Normal	NA	100%	100%	100%	100%	100%	100%

Patient # 9911	Normal control	Primary Tumor	AAH-1	AAH-2	AAH-3	AAH-4
Bases in Target Region	603600	603600	603600	603600	603600	603600
Read Length	150	150	150	150	150	150
Bases Sequenced (Filtered)	1277204700	1032937200	964094400	856384200	869483400	1097011200
Bases Mapped to Genome (filtered)	703444500	621909150	605961750	544226250	503424300	683383350
Percent Mapped to Genome	55%	60%	63%	64%	58%	62%
Bases Mapped to ROI	311161700	306277843	305562028	275650036	233775066	288929883
Percent Mapped to ROI	44%	49%	50%	51%	46%	42%
Targeted bases with at least 10 reads	579576	582314	580387	577884	576556	579264
Targeted bases with at least 10 reads (%)	96%	96%	96%	96%	96%	96%
Targeted bases with at least 10 distinct reads	578523	582094	579496	575775	573285	578445
Targeted bases with at least 10 distinct reads (%)	96%	96%	96%	95%	95%	96%
Average Raw Coverage	513	506	505	455	387	478
Average High Quality Coverage	501	495	494	445	378	467
Effective Coverage	0.97	0.97	0.97	0.97	0.96	0.97
SNPs in Tumor (> 0.4, Cov > 20 in both)	NA	96	94	104	91	92
Present in Normal	NA	96	94	104	91	92
Percent Present in Normal	NA	100%	100%	100%	100%	100%

Patient # 6709	Normal control	Primary Tumor	AAH-1	AAH-2	AAH-3	AAH-4
Bases in Target Region	603600	603600	603600	603600	603600	603600
Read Length	150	150	150	150	150	150
Bases Sequenced (Filtered)	1124090100	1175856300	1019832300	967581600	890787600	707739000
Bases Mapped to Genome (filtered)	417146700	503757300	460161900	487647750	401669550	280243200
Percent Mapped to Genome	37%	43%	45%	50%	45%	40%
Bases Mapped to ROI	213127969	257202604	228867219	233622503	209164309	151147816
Percent Mapped to ROI	51%	51%	50%	48%	52%	54%
Targeted bases with at least 10 reads	576152	576831	575950	576441	575802	573221
Targeted bases with at least 10 reads (%)	95%	96%	95%	96%	95%	95%
Targeted bases with at least 10 distinct reads	575576	576346	575302	575953	575105	568963
Targeted bases with at least 10 distinct reads (%)	95%	95%	95%	95%	95%	94%
Average Raw Coverage	354	426	380	387	347	251
Average High Quality Coverage	346	416	372	379	339	244
Effective Coverage	0.98	0.98	0.98	0.98	0.98	0.97
SNPs in Tumor (> 0.4, Cov > 20 in both)	NA	116	111	112	113	112
Present in Normal	NA	116	111	112	113	112
Percent Present in Normal	NA	100%	100%	100%	100%	100%

Patient # 4325	Normal control	Primary Tumor	AAH-1	AAH-2	AAH-3	AAH-4	AAH-5
Bases in Target Region	603600	603600	603600	603600	603600	603600	603600
Read Length	150	150	150	150	150	150	150
Bases Sequenced (Filtered)	1121971500	1188615000	1284865500	1369085700	1200032700	903749700	1029425100
Bases Mapped to Genome (filtered)	559258800	597056850	541425750	597324450	536677050	358960050	340215000
Percent Mapped to Genome	50%	50%	42%	44%	45%	40%	33%
Bases Mapped to ROI	264997285	278122625	283155431	322679836	269759552	184357339	168925868
Percent Mapped to ROI	47%	47%	52%	54%	50%	51%	50%
Targeted bases with at least 10 reads	577717	577656	576640	576744	577708	574193	573602
Targeted bases with at least 10 reads (%)	96%	96%	96%	96%	96%	95%	95%
Targeted bases with at least 10 distinct reads	577540	577325	576187	576231	576658	572753	573026
Targeted bases with at least 10 distinct reads (%)	96%	96%	95%	95%	96%	95%	95%
Average Raw Coverage	439	461	470	535	446	306	280
Average High Quality Coverage	430	452	457	520	438	299	273
Effective Coverage	0.98	0.98	0.97	0.97	0.98	0.98	0.98
SNPs in Tumor (> 0.4, Cov > 20 in both)	NA	93	94	92	94	93	94
Present in Normal	NA	93	94	92	94	93	94
Percent Present in Normal	NA	100%	100%	100%	100%	100%	100%

Patient # 7074	Normal control	Primary Tumor	AAH-1	AAH-2	AAH-3
Bases in Target Region	603600	603600	603600	603600	603600
Read Length	150	150	150	150	150
Bases Sequenced (Filtered)	1145239200	1170512700	1246455900	1223456400	1056791700
Bases Mapped to Genome (filtered)	495477300	566299650	664005450	510648900	358480800
Percent Mapped to Genome	43%	48%	53%	42%	34%
Bases Mapped to ROI	274811396	285286759	337458161	261862300	196440618
Percent Mapped to ROI	55%	50%	51%	51%	55%
Targeted bases with at least 10 reads	576512	576534	577667	576469	574260
Targeted bases with at least 10 reads (%)	96%	96%	96%	96%	95%
Targeted bases with at least 10 distinct reads	575539	575910	577045	575475	572771
Targeted bases with at least 10 distinct reads (%)	95%	95%	96%	95%	95%
Average Raw Coverage	456	474	560	435	326
Average High Quality Coverage	440	460	548	426	318
Effective Coverage	0.97	0.97	0.98	0.98	0.97
SNPs in Tumor (> 0.4, Cov > 20 in both)	NA	145	129	129	128
Present in Normal	NA	145	129	129	128
Percent Present in Normal	NA	100%	100%	100%	100%

	Normal controls	AAH
**Average High Quality Coverage**	403X	415X

Supplementary Table 2. Sequencing statistics for AAH samples.

	# of mutations
Indels	4
Splice site	2
Nonsense	1
Nonsynonymous coding	48
**Total**	**55**

mutations per lesion
**2.2**

Supplementary Table 3. Sequencing analysis of AAH lesions. A. Summary of nonsynonymous mutations and mutation frequency.

Found in this study			In COSMIC		
Gene	Amino Acid	Type	Amino Acid	Type	Tissue
ALK	311R>H	Nonsynonymous	311R>H/L	Nonsynonymous	Lung/Brain
ARID1A	1593R>W	Nonsynonymous	1593R>L	Nonsynonymous	Colon
BRAF	469G>A	Nonsynonymous	469G>A/R/S/L/V/C	Nonsynonymous	Lung/Haematopoietic
					Colon/Skin/Thyroid
					Cervix/CNS
BRAF	594D>G	Nonsynonymous	594D>G/N/H/A/V/E/K	Nonsynonymous	Lung/Colon/Skin
			D594_T599del	Deletion	Haematopoietic/CNS
BRAF	601K>E	Nonsynonymous	601K>E/I/N/K/L/Q/R	Nonsynonymous	Lung/Skin/Thyroid/Ovary/Colon/ Haematopoietic/Urinary tract
			K601del	Deletion	
EGFR	324R>H	Nonsynonymous	324R>L	Nonsynonymous	CNS
ERBB2	769D>Y	Nonsynonymous	769D>Y/H/N	Nonsynonymous	Lung/Stomach
					Head and neck, Breast/Urinary tract
ERBB2	777V>L	Nonsynonymous	777V>L/MA	Nonsynonymous	Lung/Colon/Breast/CNS
			G776_V777insVC V777_G778insCG	Insertion	
FBXW7	224R>X	Nonsense	224R>X	Nonsense	Lung/Colon/Endometrium
			224R>Q	Nonsynonymous	
FGFR4	356R>H	Nonsynonymous	356R>H	Nonsynonymous	Colon
HNF1A	179H>N	Nonsynonymous	H179fs*9	Insertion	Liver
KRAS	12G>C	Nonsynonymous	12G>C/R/N/I/L/Y/E/V/ A/W/V/F/S	Nonsynonymous	Lung/Colon/Pancreas
					Ovary/Biliary tract
MAML1	190R>H	Nonsynonymous	190R>H	Nonsynonymous	Ovary
TP53	279G>E	Nonsynonymous	279G>R/W	Nonsynonymous	Lung/Colon/Urinary tract
			p.P278_G279in	Insertion	Ovary/Head and neck/Breast

Supplementary Table 4. Sequencing analysis of AAH lesions. List of mutations found in AAH lesions that have been previously described in association with cancer.

Gene	% of mutated in lung (by COSMIC)
IKZF1	0.1%
AKT1	0.4%
GNAQ	0.6%
HNF1A	1.2%
ABL1	1.3%
FGFR4	1.6%
MAML1	1.7%
PTPN11	1.8%
FLT3	1.8%
EZH2	1.8%
TNFAIP3	1.9%

Supplementary Table 5. Sequencing analysis of AAH lesions. Mutations in genes that are rarely implicated in lung adenocarcinoma.

Most mutated genes				
**Gene**	**Amino Acid**	**# of AAH lesions**	**% of AAH lesions**	**# of patients**
**BRAF**	469G>A	4	16%	4
	594D>G			
	601K>E			
**ARID1B**	In-frame deletion	4	16%	2
	1827R>H			
	1835A>T			
	165G>A			
**MAML1**	190R>H	3	12%	2
	753G>S			
	982G>V			
**EGFR**	In-frame deletion	3	12%	2
	324R>H			
**ABL**	997P>S	2	8%	2
	608R>C			
**ATM**	Splice site	2	8%	2
	28E>V			
**MED12**	1253E>Q	2	8%	2
	1212Q>R			
**RET**	417R>H	2	8%	2
	Splice site			
**ROS1**	1895G>V	2	8%	2
	1601A>D			

Supplementary Table 6. Sequencing analysis of AAH lesions. List of most frequently mutated genes among 25 AAH lesions from 6 patients.

Adenocarcinoma In Situ

Patient # 9450	Normal control	Zone 1	Zone 2	Zone 3
Bases in Target Region	603600	603600	603600	603600
Read Length	150	150	150	150
Bases Sequenced (Filtered)	1220679600	1192994700	1064838000	1860708300
Bases Mapped to Genome (filtered)	730832100	716853750	682192950	1205893800
Percent Mapped to Genome	60%	60%	64%	65%
Bases Mapped to ROI	150014720	154757726	155585151	240591144
Percent Mapped to ROI	21%	22%	23%	20%
Targeted bases with at least 10 reads	575257	576131	575555	581523
Targeted bases with at least 10 reads (%)	95%	95%	95%	96%
Targeted bases with at least 10 distinct reads	575165	575953	575305	581373
Targeted bases with at least 10 distinct reads (%)	95%	95%	95%	96%
Average Raw Coverage	248.7	256.4	257.7	398.2
Average High Quality Coverage	240.4	247.5	248.8	381.0
Effective Coverage	0.97	0.97	0.97	0.96
SNPs in Tumor (> 0.4, Cov > 20 in both)	NA	96	99	99
Present in Normal	NA	96	99	99
Percent Present in Normal	NA	100%	100%	100%

Patient # 3983	Normal control	Zone 1	Zone 2	Zone 3
Bases in Target Region	603600	603600	603600	603600
Read Length	150	150	150	150
Bases Sequenced (Filtered)	1302508500	1329149700	903294000	1405298700
Bases Mapped to Genome (filtered)	669664200	578685750	455576850	591453150
Percent Mapped to Genome	51%	44%	50%	42%
Bases Mapped to ROI	151305270	113465224	96881665	108788771
Percent Mapped to ROI	23%	20%	21%	18%
Targeted bases with at least 10 reads	572262	566809	566433	568064
Targeted bases with at least 10 reads (%)	95%	94%	94%	94%
Targeted bases with at least 10 distinct reads	572072	566502	565950	567963
Targeted bases with at least 10 distinct reads (%)	95%	94%	94%	94%
Average Raw Coverage	252.4	189.3	161.6	181.3
Average High Quality Coverage	243.6	182.4	158.3	177.6
Effective Coverage	0.96	0.96	0.98	0.98
SNPs in Tumor (> 0.4, Cov > 20 in both)	NA	125	123	118
Present in Normal	NA	125	123	118
Percent Present in Normal	NA	100%	100%	100%

Patient # 0429	Normal control	Zone 1	Zone 2	Zone 3
Bases in Target Region	603600	603600	603600	603600
Read Length	150	150	150	150
Bases Sequenced (Filtered)	1584232500	1336380000	1202961900	1560287100
Bases Mapped to Genome (filtered)	847134000	816027150	691919250	790590150
Percent Mapped to Genome	53%	61%	58%	51%
Bases Mapped to ROI	169780657	172354217	128582669	123249785
Percent Mapped to ROI	20%	21%	19%	16%
Targeted bases with at least 10 reads	575166	575919	573234	572151
Targeted bases with at least 10 reads (%)	95%	95%	95%	95%
Targeted bases with at least 10 distinct reads	574972	575055	573026	572077
Targeted bases with at least 10 distinct reads (%)	95%	95%	95%	95%
Average Raw Coverage	282.1	285.6	213.6	204.9
Average High Quality Coverage	269.2	274.3	204.0	194.8
Effective Coverage	0.95	0.96	0.96	0.95
SNPs in Tumor (> 0.4, Cov > 20 in both)	NA	107	136	132
Present in Normal	NA	107	136	132
Percent Present in Normal	NA	100%	100%	100%

Patient # 3916	Normal control	Zone 1	Zone 2	Zone 3
Bases in Target Region	603600	603600	603600	603600
Read Length	150	150	150	150
Bases Sequenced (Filtered)	1022771100	976716600	1161665100	988319400
Bases Mapped to Genome (filtered)	389597250	530754300	515854200	211730250
Percent Mapped to Genome	38%	54%	44%	21%
Bases Mapped to ROI	69742038	91813472	89798773	38706527
Percent Mapped to ROI	18%	17%	17%	18%
Targeted bases with at least 10 reads	564601	567627	567612	551920
Targeted bases with at least 10 reads (%)	94%	94%	94%	91%
Targeted bases with at least 10 distinct reads	564395	565893	567192	549591
Targeted bases with at least 10 distinct reads (%)	94%	94%	94%	91%
Average Raw Coverage	116.4	153.3	150.0	64.8
Average High Quality Coverage	113.9	150.1	147.6	63.5
Effective Coverage	0.98	0.98	0.98	0.98
SNPs in Tumor (> 0.4, Cov > 20 in both)	NA	99	99	94
Present in Normal	NA	99	99	94
Percent Present in Normal	NA	100%	100%	100%

Patient # 9309	Normal control	Zone 1	Zone 2	Zone 3
Bases in Target Region	603600	603600	603600	603600
Read Length	150	150	150	150
Bases Sequenced (Filtered)	1237539900	1052806800	1186132500	843049800
Bases Mapped to Genome (filtered)	1135824300	420859950	637284600	392896650
Percent Mapped to Genome	92%	40%	54%	47%
Bases Mapped to ROI	550368179	267671711	394970831	237215690
Percent Mapped to ROI	48%	64%	62%	60%
Targeted bases with at least 10 reads	589033	564447	575545	570398
Targeted bases with at least 10 reads (%)	98%	94%	95%	94%
Targeted bases with at least 10 distinct reads	588337	501366	572098	557840
Targeted bases with at least 10 distinct reads (%)	97%	83%	95%	92%
Average Raw Coverage	910	439	648	389
Average High Quality Coverage	880	428	632	380
Effective Coverage	0.97	0.97	0.98	0.98
SNPs in Tumor (> 0.4, Cov > 20 in both)	NA	77	121	100
Present in Normal	NA	77	121	100
Percent Present in Normal	NA	100%	100%	100%

	Normal controls	AIS
**Average High Quality Coverage**	349X	258X

Supplementary Table 7. Sequencing statistics for AIS samples.

Minimally Invasive Adenocarcinoma

Patient # 2035	Normal control	Zone 1	Zone 2	Zone 3	Zone 4
Bases in Target Region	603600	603600	603600	603600	603600
Read Length	150	150	150	150	150
Bases Sequenced (Filtered)	736247400	627886500	578024700	782879400	748570800
Bases Mapped to Genome (filtered)	320198850	270201900	277781100	402252900	359062350
Percent Mapped to Genome	43%	43%	48%	51%	48%
Bases Mapped to ROI	165655297	151605704	143965939	202168866	185706669
Percent Mapped to ROI	52%	56%	52%	50%	52%
Targeted bases with at least 10 reads	573251	572951	571706	574730	574226
Targeted bases with at least 10 reads (%)	95%	95%	95%	95%	95%
Targeted bases with at least 10 distinct reads	572721	570460	568706	573267	573115
Targeted bases with at least 10 distinct reads (%)	95%	95%	94%	95%	95%
Average Raw Coverage	275	251	238	334	308
Average High Quality Coverage	272	248	235	324	303
Effective Coverage	0.99	0.99	0.98	0.97	0.99
SNPs in Tumor (> 0.4, Cov > 20 in both)	NA	111	114	117	109
Present in Normal	NA	111	114	117	109
Percent Present in Normal	NA	100%	100%	100%	100%

Patient # 1061	Normal control	Zone 1	Zone 2	Zone 3	Zone 4
Bases in Target Region	603600	603600	603600	603600	603600
Read Length	150	150	150	150	150
Bases Sequenced (Filtered)	1266284100	1416911700	1482048000	1293621300	1258219200
Bases Mapped to Genome (filtered)	537972300	704701800	655845300	698957550	529250100
Percent Mapped to Genome	42%	50%	44%	54%	42%
Bases Mapped to ROI	300491220	388919161	360429489	387519316	285209827
Percent Mapped to ROI	56%	55%	55%	55%	54%
Targeted bases with at least 10 reads	577663	580150	578555	579529	575847
Targeted bases with at least 10 reads (%)	96%	96%	96%	96%	95%
Targeted bases with at least 10 distinct reads	575915	578344	574996	577927	573894
Targeted bases with at least 10 distinct reads (%)	95%	96%	95%	96%	95%
Average Raw Coverage	500	643	598	640	473
Average High Quality Coverage	486	625	581	625	459
Effective Coverage	0.97	0.97	0.97	0.98	0.97
SNPs in Tumor (> 0.4, Cov > 20 in both)	NA	92	97	108	96
Present in Normal	NA	92	97	108	96
Percent Present in Normal	NA	100%	100%	100%	100%

Patient # 6610	Normal control	Zone 1	Zone 2	Zone 3	Zone 4
Bases in Target Region	603600	603600	603600	603600	603600
Read Length	150	150	150	150	150
Bases Sequenced (Filtered)	1082588700	1056920400	1052701200	1087895400	1343589600
Bases Mapped to Genome (filtered)	620848800	433523550	461219400	569649600	685253700
Percent Mapped to Genome	57%	41%	44%	52%	51%
Bases Mapped to ROI	316416765	224836878	228814708	261469391	302795553
Percent Mapped to ROI	51%	52%	50%	46%	44%
Targeted bases with at least 10 reads	579587	576591	576912	578566	578456
Targeted bases with at least 10 reads (%)	96%	96%	96%	96%	96%
Targeted bases with at least 10 distinct reads	579134	575677	576155	577934	578261
Targeted bases with at least 10 distinct reads (%)	96%	95%	95%	96%	96%
Average Raw Coverage	524	371	378	432	501
Average High Quality Coverage	513	362	370	420	489
Effective Coverage	0.98	0.98	0.98	0.97	0.98
SNPs in Tumor (> 0.4, Cov > 20 in both)	NA	119	118	128	121
Present in Normal	NA	119	118	128	121
Percent Present in Normal	NA	100%	100%	100%	100%

Patient # 6992	Normal control	Zone 1	Zone 2	Zone 3	Zone 4
Bases in Target Region	603600	603600	603600	603600	603600
Read Length	150	150	150	150	150
Bases Sequenced (Filtered)	1086844200	875968800	1114887000	723695100	949677300
Bases Mapped to Genome (filtered)	656897850	502475100	821004300	459004050	541089300
Percent Mapped to Genome	60%	57%	74%	63%	57%
Bases Mapped to ROI	341014053	279194556	418903182	206450411	249873620
Percent Mapped to ROI	52%	56%	51%	45%	46%
Targeted bases with at least 10 reads	576826	577743	584860	578385	579630
Targeted bases with at least 10 reads (%)	96%	96%	97%	96%	96%
Targeted bases with at least 10 distinct reads	576423	570889	584248	578013	579535
Targeted bases with at least 10 distinct reads (%)	95%	95%	97%	96%	96%
Average Raw Coverage	564	463	691	342	413
Average High Quality Coverage	552	455	673	336	406
Effective Coverage	0.98	0.98	0.97	0.98	0.98
SNPs in Tumor (> 0.4, Cov > 20 in both)	NA	102	117	126	125
Present in Normal	NA	102	117	126	125
Percent Present in Normal	NA	100%	100%	100%	100%

Patient # 4845	Normal control	Zone 1	Zone 2	Zone 3	Zone 4
Bases in Target Region	603600	603600	603600	603600	603600
Read Length	150	150	150	150	150
Bases Sequenced (Filtered)	1012467000	900561900	839101800	946932900	1042920600
Bases Mapped to Genome (filtered)	735791700	605086800	589477350	711672450	794575800
Percent Mapped to Genome	73%	67%	70%	75%	76%
Bases Mapped to ROI	370136749	301309780	298528212	333223400	363446038
Percent Mapped to ROI	50%	50%	51%	47%	46%
Targeted bases with at least 10 reads	583580	583042	581816	589368	589045
Targeted bases with at least 10 reads (%)	97%	97%	96%	98%	98%
Targeted bases with at least 10 distinct reads	583450	582853	581737	589190	588985
Targeted bases with at least 10 distinct reads (%)	97%	97%	96%	98%	98%
Average Raw Coverage	611	496	493	548	598
Average High Quality Coverage	601	487	484	539	588
Effective Coverage	0.98	0.98	0.98	0.98	0.98
SNPs in Tumor (> 0.4, Cov > 20 in both)	N/A	98	100	113	116
Present in Normal	N/A	98	100	113	116
Percent Present in Normal	N/A	100%	100%	100%	100%

	Normal controls	MIA
Average High Quality Coverage	485X	450X

Supplementary Table 8. Sequencing statistics for MIA samples.

	# of unique mutations	
	**AIS**	**MIA**
Indels	1	4
Splice site	3	2
Nonsense	0	1
Nonsynonymous coding	17	23
**Total**	**21**	**30**

Supplementary Table 9. Sequencing analysis of AIS and MIA cohorts. Summary of nonsynonymous unique mutations found in both cohorts.

	# of mutated genes	# of mutations (all zones)	mutations per zone	mutations per patient
**AIS**	19	31	2.1	6.2
**MIA**	19	54	2.7	10.8

Supplementary Table 10. Sequencing analysis of AIS and MIA cohorts. Table demonstrates the number of effected genes, total mutations number and mutations rate per zone and per patient in each group.

Most mutated genes in AIS					
AIS			MIA		
Gene	Amino Acid	# of patients	Gene	Amino Acid	# of patients
ARID1A	1396G>A<br>1528R>Q	2	EGFR	In-frame deletion<br>In-frame insertion<br>790T>M<br>858L>R	4
			TP53	245G>S<br>204E>D<br>154G>V/D	3
			ATRX	929Q>E<br>183K>E	2
			CTNNB1	535R>Q<br>Splice site	2
			MED12	709E>K<br>784R>H	2

Supplementary Table 11. Sequencing analysis of AIS and MIA cohorts. Most frequently mutated genes in AIS and MIA tumors.

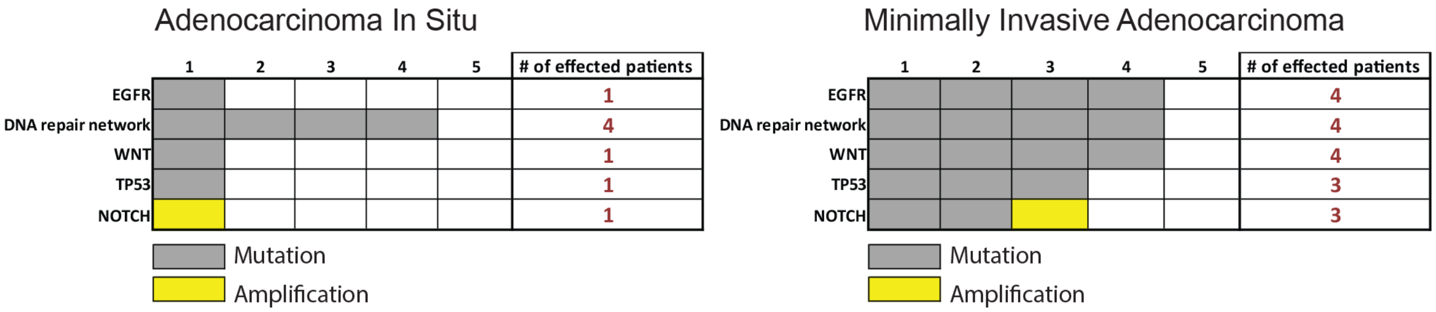

Supplementary Table 12. Summary of genes and potentially affected pathways differentially altered in AIS versus MIA tumors.

Recurrent mutations in MIA		
**Gene**	**Amino Acid**	**# of patients**
EGFR	858L>R	2
TP53	154G>V/D	2

Supplementary Table 13. Sequencing analysis of AIS and MIA cohorts. Recurrent mutations in MIA patients.

Genes mutated in boht: AIS and MIA
APC
CCND1
EGFR
IGF1R
TET2
TP53

Supplementary Table 14. Sequencing analysis of AIS and MIA cohorts. List of genes that found to be mutated in both AIS and MIA patients.

AIS					
Found in this study			In COSMIC		
Gene	Amino Acid	Type	Amino Acid	Type	Tissue
APC	1406Q>H	Nonsynonymous	1406Q>H	Nonsynonymous	Colon/CNS/Skin
			1406Q>X	Nonsense	
			Q1406fs*11	Insertion	
			Q1406fs*9	Deletion	
ARID1A	1528R>Q	Nonsynonymous	1528R>X	Nonsense	Endometrium/Ovary Haematopoietic
ATM	250R>X	Nonsense	250R>X	Nonsense	Colon/Kidney Endometrium
EGFR	858L>R	Nonsynonymous	858L>R/M/K/Q/A/G/P/ V/W	Nonsynonymous	Lung/Breast Upper aerodigestive tract
GNAS	232R>C	Nonsynonymous	232R>H	Nonsynonymous	Endometrium
KRAS	12G>C	Nonsynonymous	12G>C/R/N/I/L/Y/E/V/ A/W/V/F/S	Nonsynonymous	Lung/Colon/Pancreas Ovary/Biliary tract
MSH6	922R>Q	Nonsynonymous	922R>Q	Nonsynonymous	Colon
			922R>X	Nonsense	
PBRM1	1565W>X	Nonsynonymous	L1565fs*11	Deletion	Kidney
			L1565fs*>19	Insertion	
PBRM1	160E>K	Nonsense	160E>K	Nonsense	Kidney
SF3B1	700K>E	Nonsynonymous	700K>E	Nonsynonymous	Haematopoietic CNS/Breast/Pancreas
TSC2	354Q>R	Nonsynonymous	354Q>H	Nonsynonymous	Lung

MIA					
Found in this study			In COSMIC		
Gene	Amino Acid	Type	Amino Acid	Type	Tissue
APC	1444Q>R	Nonsynonymous	1444Q>R/P	Nonsynonymous	Colon/Pancreas/
			1444Q>X	Nonsense	
			Q1444fs*29	Deletion	
BRAF	581N>S	Nonsynonymous	581N>S/T/I/Y	Nonsynonymous	Lung/Colon/Ovary/Skin
CTNNB1	535R>Q	Nonsynonymous	535R>Q	Nonsynonymous	Endometrium
EGFR	858L>R	Nonsynonymous	858L>R/M/K/Q/A/G/P/ V/W	Nonsynonymous	Lung/Breast Upper aerodigestive tract
EGFR	790T>M	Nonsynonymous	790T>M/A	Nonsynonymous	Lung Oesophagus
MED12	784R>H	Nonsynonymous	784R>H	Nonsynonymous	Haematopoietic
NOTCH3	1700D>Y	Nonsynonymous	1700D>Y	Nonsynonymous	Lung
PTEN	301D>N	Nonsynonymous	D301fs*6	Deletion	Endometrium
TP53	245G>S	Nonsynonymous	245G>S/R	Nonsynonymous	Colon/Breast/OvaryLiver/ Urinary tract/ CNS/Pancreas
			G245delG	Deletion	
			G245fs*17	Deletion	
			G245fs*14	Deletion	
TP53	154G>V	Nonsynonymous	154G>S/C/I/D/A/V	Nonsynonymous	Lung/Cervix/Colon/ Adrenal gland/Skin/Upper aerodigestive tract/Pancreas/Liver/ Breast/Prostate
			G154fs*14	Deletion	
			G154fs*16	Deletion	
			p.G154fs*22	Deletion	
			G154_R156delGTR	Deletion	
			G154fs*27	Insersion	

Supplementary Table 15. Sequencing analysis of AIS and MIA cohorts. List of mutations found in either AIS or MIA patients that have been previously observed in solid tumors (including lung) and hematopoietic malignancies.

AIS	
**Gene**	**% of mutated in lung (by COSMIC)**
**ABL1**	**1.3%**
**CCND1**	**0.7%**
**GATA1**	**1.2%**
**GNAS**	**1.2%**
**KIT**	**1.5%**
**TET2**	**1.7%**

MIA	
**Gene**	**% of mutated in lung (by COSMIC)**
**CCND1**	**0.7%**
**MPL**	**1.0%**
**TET2**	**1.7%**

Supplementary Table 16. Sequencing analysis of AIS and MIA cohorts. Mutations in genes that are rarely implicated in lung adenocarcinoma.

Patient ID	Age	Race	Gender	Hispanic or Latino (Y/N)	Smoking history
**9450**	64	White	Male	No	Yes
**3983**	72	Black	Female	No	No
**0429**	84	White	Female	No	Yes
**3916**	70	White	Male	No	No
**9309**	45	Black	Female	No	No
**2035**	41	White	Female	No	No
**1061**	73	White	Male	No	Yes
**6610**	71	Black	Male	No	Yes
**6992**	61	White	Female	No	Yes
**4845**	73	White	Male	No	Yes
**7074**	68	asian	Male	No	Yes
**4325**	62	White	Female	No	Yes
**6709**	62	White	Female	No	Yes
**9911**	74	White	Female	No	Yes
**7484**	64	White	Male	No	No
**7337**	76	OTHER - from Bermuda	Male	No	Yes

Supplementary Table 17. Socio-demographic data of patients used in this study. Patient's race and ethnicity data, age and smoking history.

Atypical Adenomatous Hyperplasia

Patient # 7337

Specimen ID	Histologic samples within the same lesion	Sample type	Stage*
54560	AAH-1	Atypical Adenomatous Hyperplasia	
54561	AAH-2		
54562	AAH-3		
54563	AAH-4		
54564	Primary Tumor	Adenocarcinoma	T1N0MX
**54565**	**Lymph Node**		

Patient # 7484

Specimen ID	Histologic samples within the same lesion	Sample type	Stage*
54555	AAH-1	Atypical Adenomatous Hyperplasia	
54672	AAH-2		
54557	AAH-3		
54554	AAH-4		
54556	AAH-5		
54558	Primary Tumor	Adenocarcinoma	T4N2M0
**54559**	**Lymph Node**		

Patient # 9911

Specimen ID	Histologic samples within the same lesion	Sample type	Stage*
54548	AAH-1	Atypical Adenomatous Hyperplasia	
54550	AAH-2		
54551	AAH-3		
54552	AAH-4		
54553	Primary Tumor	Adenocarcinoma	T1N0M0
**54549**	**Normal Lung**		

Patient # 6709

Specimen ID	Histologic samples within the same lesion	Sample type	Stage*
54542	AAH-1	Atypical Adenomatous Hyperplasia	
54543	AAH-2		
54544	AAH-3		
54545	AAH-4		
54546	Primary Tumor	Adenocarcinoma	T2N0M0
**54547**	**Lymph Node**		

Patient # 4325

Specimen ID	Histologic samples within the same lesion	Sample type	Stage*
54535	AAH-1	Atypical Adenomatous Hyperplasia	
54537	AAH-2		
54538	AAH-3		
54539	AAH-4		
54540	AAH-5		
54536	Primary Tumor	Adenocarcinoma	T1N0M0
**54541**	**Lymph node**		

Patient # 7074

Specimen ID	Histologic samples within the same lesion	Sample type	Stage*
54520	AAH-1	Atypical Adenomatous Hyperplasia	
54522	AAH-2		
54524	AAH-3		
54532	Primary Tumor	Adenocarcinoma	T2N0M0
**54533**	**Lymph node**		

*For the AAHs, the provided staging information is for the corresponding invasive lung adenocarcinomas

Supplementary Table 18. List of AAH samples collected for this study and histopathological diagnostics.

Adenocarcinoma in Situ

Patient # 9450

Specimen ID	Histologic progression within the same lesion	Tumor type	Histopathologic Diagnosis	Stage
50833	Tumor Zone 1	Bronchioloalveolar adenocarcinoma	Adenocarcinoma in Situ	TisN0M0
50835	Tumor Zone 2			
50836	Tumor Zone 3			
**50834**	**Control Lymph node**			

Patient # 3983

Sample number	Histologic progression within the same lesion	Tumor type	Histopathologic Diagnosis	Stage
50838	Tumor Zone 1	Bronchioloalveolar adenocarcinoma	Adenocarcinoma in Situ	TisN0M0
50841	Tumor Zone 2			
50842	Tumor Zone 3			
**50837**	**Control Lymph node**			

Patient # 0429

Sample number	Histologic progression within the same lesion	Tumor type	Histopathologic Diagnosis	Stage
50951	Tumor Zone 1	Bronchioloalveolar adenocarcinoma	Adenocarcinoma in Situ	TisN0M0
50952	Tumor Zone 2			
50953	Tumor Zone 3			
**50950**	**Control Lymph node**			

Patient # 3916

Sample number	Histologic progression within the same lesion	Tumor type	Histopathologic Diagnosis	Stage
50954	Tumor Zone 1	Bronchioloalveolar adenocarcinoma	Adenocarcinoma in Situ	TisN0M0
50955	Tumor Zone 2			
50956	Tumor Zone 3			
**50997**	**Control Lymph node**			

Patient # 9309

Sample number	Histologic progression within the same lesion	Tumor type	Histopathologic Diagnosis	Stage
52274	Tumor Zone 1	Bronchioloalveolar adenocarcinoma	Adenocarcinoma in Situ	TisN0M0
52275	Tumor Zone 2			
52276	Tumor Zone 3			
**52277**	**Control Lymph node**			

Supplementary Table 19. List of AIS samples collected for this study and histopathological diagnostics.

Minimally invasive adenocarcinoma

Patient # 2035

Specimen ID	Histologic progression within the same lesion	Tumor type	Histopathologic Diagnosis	Stage
52283	Tumor Zone 1	Bronchioloalveolar adenocarcinoma	Minimally invasive adenocarcinoma	T1N0M0
52284	Tumor Zone 2			
52285	Tumor Zone 3			
52286	Tumor Zone 4			
**52292**	**Control Lymph node**			

Patient # 1061

Specimen ID	Histologic progression within the same lesion	Tumor type	Histopathologic Diagnosis	Stage
52287	Tumor Zone 1	Bronchioloalveolar adenocarcinoma	Minimally invasive adenocarcinoma	T1N0M0
52288	Tumor Zone 2			
52289	Tumor Zone 3			
52290	Tumor Zone 4			
**52291**	**Control Lymph node**			

Patient # 4845

Specimen ID	Histologic progression within the same lesion	Tumor type	Histopathologic Diagnosis	Stage
53590	Tumor Zone 1	Bronchioloalveolar adenocarcinoma	Minimally invasive adenocarcinoma	T1N0M0
53591	Tumor Zone 2			
53592	Tumor Zone 3			
53593	Tumor Zone 4			
**53594**	**Control Lymph node**			

Patient # 6992

Specimen ID	Histologic progression within the same lesion	Tumor type	Histopathologic Diagnosis	Stage
52600	Tumor Zone 1	Bronchioloalveolar adenocarcinoma	Minimally invasive adenocarcinoma	T1N0M0
52601	Tumor Zone 2			
52602	Tumor Zone 3			
52603	Tumor Zone 4			
**52604**	**Control Lymph node**			

Patient # 6610

Specimen ID	Histologic progression within the same lesion	Tumor type	Histopathologic Diagnosis	Stage
52589	Tumor Zone 1	Bronchioloalveolar adenocarcinoma	Minimally invasive adenocarcinoma	T1N0M0
52590	Tumor Zone 2			
52597	Tumor Zone 3			
52599	Tumor Zone 4			
**52586**	**Control Lymph node**			

Supplementary Table 20. List of MIA samples collected for this study and histopathological diagnostics.

Atypical Adenomatous Hyperplasia

Patient # 7337

Histologic samples within the same lesion	Gene	Mutation Type	Consequence	Amino Acid (protein)	Mutant reads	Distinct Total Reads	Distinct Mutant Reads
AAH-1	ALK	Substitution	Nonsynonymous coding	311R>H	4%	250	10
	ARID1B	Deletion	In-frame deletion	NA	3%	590	17
	EZH2	Substitution	Nonsynonymous coding	185D>H	3%	276	7
	IGF1R	Substitution	Nonsynonymous coding	895R>W	3%	506	15
	MAML1	Substitution	Nonsynonymous coding	190R>H	6%	367	21
	MAML1	Substitution	Nonsynonymous coding	753G>S	3%	382	12
	PTCH1	Substitution	Nonsynonymous coding	1122M>K	4%	314	12
AAH-2	BRAF	Substitution	Nonsynonymous coding	601K>E	2%	563	12
AAH-3	EGFR	Deletion	In-frame deletion	NA	2%	457	7
	TP53	Substitution	Nonsynonymous coding	279G>E	3%	225	6
AAH-4	ARID1B	Substitution	Nonsynonymous coding	1827R>H	2%	720	15
	EGFR	Deletion	In-frame deletion	NA	2%	290	6
	TP53	Substitution	Nonsynonymous coding	279G>E	17%	766	128
Primary Tumor	EGFR	Deletion	In-frame deletion	NA	9%	882	80
	TP53	Substitution	Nonsynonymous coding	279G>E	20%	593	117

Patient # 7484

Histologic samples within the same lesion	Gene	Mutation Type	Consequence	Amino Acid (protein)	Mutant reads	Distinct Total Reads	Distinct Mutant Reads
AAH-1	No somatic sequence alterations found						
AAH-2	No somatic sequence alterations found						
AAH-3	BRAF	Substitution	Nonsynonymous coding	469G>A	3%	316	10
AHH-4	No somatic sequence alterations found						
AAH-5	No somatic sequence alterations found						
Primary Tumor	ATM	Substitution	Nonsynonymous coding	2455L>M	35%	140	49
	ATRX	Substitution	Nonsynonymous coding	929Q>E	4%	112	4
	BRAF	Substitution	Nonsynonymous coding	466G>V	20%	176	35
	ERBB2	Substitution	Nonsynonymous coding	243G>S	25%	399	101
	IKZF1	Substitution	Nonsynonymous coding	210Y>C	22%	246	54
	NRAS	Substitution	Nonsynonymous coding	13G>R	20%	490	100
	STK11	Substitution	Splice site donor	NA	41%	230	94

Patient # 7484

Gene	Folds	Amplification/Deletion
MET	3.1	Amplification
CDKN2A	NA	Deletion

Patient # 9911

Histologic samples within the same lesion	Gene	Mutation Type	Consequence	Amino Acid (protein)	Mutant reads	Distinct Total Reads	Distinct Mutant Reads
AAH-1	BRAF	Substitution	Nonsynonymous coding	594D>G	9%	333	29
	ROS1	Substitution	Nonsynonymous coding	1895G>V	10%	544	56
AAH2	ERBB2	Substitution	Nonsynonymous coding	769D>Y	2%	259	6
	ERBB2	Substitution	Nonsynonymous coding	777V>L	3%	252	8
	ERBB4	Substitution	Nonsynonymous coding	210R>K	5%	251	12
	HNF1A	Substitution	Nonsynonymous coding	179H>N	5%	240	12
	KRAS	Substitution	Nonsynonymous coding	12G>C	6%	252	16
	MAML1	Substitution	Nonsynonymous coding	190R>H	10%	274	28
AAH-3	KRAS	Substitution	Nonsynonymous coding	12G>C	8%	197	15
	MAML1	Substitution	Nonsynonymous coding	982G>V	8%	48	4
	MLL	Substitution	Nonsynonymous coding	723S>Y	4%	130	5
AAH-4	ABL1	Substitution	Nonsynonymous coding	997P>S	12%	338	42
	ASXL1	Substitution	Nonsynonymous coding	371L>M	11%	412	45
	GNAQ	Substitution	Nonsynonymous coding	60R>T	15%	514	76
	KRAS	Substitution	Nonsynonymous coding	12G>C	8%	393	31
	MED12	Substitution	Nonsynonymous coding	1253E>Q	8%	239	18
	RET	Substitution	Nonsynonymous coding	417R>H	3%	116	4
Primary Tumor	KRAS	Substitution	Nonsynonymous coding	12G>C	13%	457	59

Patient # 6709

Histologic samples within the same lesion	Gene	Mutation Type	Consequence	Amino Acid (protein)	Mutant reads	Distinct Total Reads	Distinct Mutant Reads
AAH-1	DNMT3A	Substitution	Nonsynonymous coding	261T>M	2%	184	4
AAH-2	No somatic sequence alterations found						
AAH-3	No somatic sequence alterations found						
AAH-4	ATM	Substitution	Splice site donor	NA	7%	55	4
	FBXW7	Substitution	Nonsense	224R>X	4%	139	5
	FGFR4	Substitution	Nonsynonymous coding	356R>H	4%	105	4
	MET	Substitution	Nonsynonymous coding	965G>S	5%	86	4
	ROS1	Substitution	Nonsynonymous coding	1601A>D	14%	96	13
	TSC2	Substitution	Nonsynonymous coding	138E>K	3%	132	4
Primary Tumor	ARID1A	Substitution	Nonsynonymous coding	1551R>C	2%	183	4
	ATRX	Substitution	Nonsynonymous coding	319K>N	8%	231	19
	FGFR3	Substitution	Nonsynonymous coding	804S>L	2%	193	4
	KRAS	Substitution	Nonsynonymous coding	12G>D	12%	367	44
	NOTCH1	Substitution	Nonsynonymous coding	348A>T	3%	159	4
	PIK3CA	Substitution	Nonsynonymous coding	1047H>R	19%	526	99
	RET	Substitution	Nonsynonymous coding	867E>K	4%	111	4
	STK11	Substitution	Nonsynonymous coding	397A>V	2%	170	4
	TSHR	Substitution	Nonsynonymous coding	249S>Y	17%	525	89

Patient # 4325

Histologic samples within the same lesion	Gene	Mutation Type	Consequence	Amino Acid (protein)	Mutant reads	Distinct Total Reads	Distinct Mutant Reads
AAH-1	BRAF	Substitution	Nonsynonymous coding	469G>A	2%	347	7
	MED12	Substitution	Nonsynonymous coding	1212Q>R	3%	366	11
	PTPN11	Substitution	Nonsynonymous coding	419H>Q	3%	265	7
	TNFAIP3	Substitution	Nonsynonymous coding	129T>K	2%	773	19
AAH-2	No somatic sequence alterations found						
AAH3	ATRX	Substitution	Nonsynonymous coding	929Q>E	2%	379	8
	IKZF1	Substitution	Nonsynonymous coding	164I>M	8%	262	22
	NF1	Deletion	Frameshift	NA	10%	344	35
AAH-4	ABL1	Substitution	Nonsynonymous coding	608R>C	3%	144	4
	ARID1B	Substitution	Nonsynonymous coding	1835A>T	2%	191	4
	EGFR	Substitution	Nonsynonymous coding	324R>H	2%	172	4
	FGFR2	Substitution	Nonsynonymous coding	550N>D	4%	134	5
	FLT3	Substitution	Nonsynonymous coding	228D>N	2%	168	4
	PDGFRA	Substitution	Nonsynonymous coding	279E>K	2%	199	4
AAH5	ARID1A	Substitution	Nonsynonymous coding	1593R>W	2%	230	5
	ARID1B	Substitution	Nonsynonymous coding	165G>A	19%	42	8
	ATM	Substitution	Nonsynonymous coding	28E>V	16%	202	32
Primary Tumor	GNAS	Deletion	Frameshift	NA	6%	385	23
	NRAS	Substitution	Nonsynonymous coding	61Q>L	7%	445	31
	PDGFRA	Substitution	Nonsynonymous coding	478S>P	3%	645	18
	PDGFRA	Substitution	Nonsynonymous coding	956G>R	4%	733	27
	TP53	Substitution	Nonsynonymous coding	155T>P	4%	469	21

Patient # 7074

Histologic samples within the same lesion	Gene	Mutation Type	Consequence	Amino Acid (protein)	Mutant reads	Distinct Total Reads	Distinct Mutant Reads
AAH-1	No somatic sequence alterations found						
AAH-2	No somatic sequence alterations found						
AAH-3	AKT1	Substitution	Nonsynonymous coding	58A>V	2%	175	4
	RET	Substitution	Splice site donor	NA	3%	144	4
Primary Tumor	KRAS	Substitution	Nonsynonymous coding	12G>C	21%	467	100
	NOTCH3	Substitution	Nonsynonymous coding	558R>C	2%	191	4
	PAX5	Substitution	Nonsynonymous coding	31R>P	16%	507	80
	PTEN	Substitution	Nonsynonymous coding	252D>Y	42%	256	107
	TP53	Substitution	Nonsynonymous coding	342R>P	19%	346	65
	TP53	Substitution	Nonsynonymous coding	242C>F	18%	386	70

Supplementary Table 21. Summary of all mutations and copy number variations identified in AAH samples. Table lists mutated genes, mutations type, amino acid changes, distinct coverage and percentage of mutant reads identified.

Adenocarcinoma in Situ

Patient # 9450

Histologic progression within the same lesion	Gene	Mutation Type	Consequence	Amino Acid (protein)	Mutant reads	Distinct Total Reads	Distinct Mutant Reads
Tumor Zone 1	APC	Substitution	Nonsynonymous coding	1406Q>H	6%	300	18
	KIT	Substitution	Nonsynonymous coding	630H>Y	13%	388	49
	KRAS	Substitution	Nonsynonymous coding	12G>C	10%	278	28
	SF3B1	Substitution	Nonsynonymous coding	700K>E	12%	350	43
	TET2	Substitution	Nonsynonymous coding	1045A>D	8%	414	35
Tumor Zone 2	APC	Substitution	Nonsynonymous coding	1406Q>H	7%	305	21
	KIT	Substitution	Nonsynonymous coding	630H>Y	12%	373	44
	KRAS	Substitution	Nonsynonymous coding	12G>C	11%	331	38
	SF3B1	Substitution	Nonsynonymous coding	700K>E	18%	413	74
	TET2	Substitution	Nonsynonymous coding	1045A>D	16%	490	78
Tumor Zone 3	ARID1A	Substitution	Nonsynonymous coding	1396G>A	6%	320	18
	TP53	Substitution	Nonsense	136Q>X	10%	545	52
	KRAS	Substitution	Nonsynonymous coding	12G>C	10%	649	63
	SF3B1	Substitution	Nonsynonymous coding	700K>E	9%	753	71
	TET2	Substitution	Nonsynonymous coding	1045A>D	4%	312	12

Patient # 3983

Histologic progression within the same lesion	Gene	Mutation Type	Consequence	Amino Acid (protein)	Mutant reads	Distinct Total Reads	Distinct Mutant Reads
Tumor Zone 1	No somatic sequence alterations found						
Tumor Zone 2	No somatic sequence alterations found						
Tumor Zone 3	ARID1A	Substitution	Nonsynonymous coding	1528R>Q	3%	140	4
	ERBB4	Substitution	Nonsynonymous coding	1134V>M	2%	240	5
	IGF1R	Substitution	Nonsynonymous coding	283A>T	4%	108	4

Patient # 0429

Histologic progression within the same lesion	Gene	Mutation Type	Consequence	Amino Acid (protein)	Mutant reads	Distinct Total Reads	Distinct Mutant Reads
Tumor Zone 1	EGFR	Substitution	Nonsynonymous coding	858L>R	19%	151	28
Tumor Zone 2	EGFR	Substitution	Nonsynonymous coding	858L>R	16%	222	35
Tumor Zone 3	EGFR	Substitution	Nonsynonymous coding	858L>R	17%	169	28

Patient # 3916

Histologic progression within the same lesion	Gene	Mutation Type	Consequence	Amino Acid (protein)	Mutant reads	Distinct Total Reads	Distinct Mutant Reads
Tumor Zone 1	MET	Deletion	Frameshift	NA	4%	147	6
Tumor Zone 2	No somatic sequence alterations found						
Tumor Zone 3	ATM	Substitution	Nonsense	250R>X	5%	78	4

Patient # 9309

Histologic progression within the same lesion	Gene	Mutation Type	Consequence	Amino Acid (protein)	Mutant reads	Distinct Total Reads	Distinct Mutant Reads
Tumor Zone 1	ABL1	Substitution	Nonsynonymous coding	1101A>V	6%	72	4
	CCND1	Substitution	Nonsynonymous coding	169P>S	3%	157	4
	PBRM1	Substitution	Nonsense	1565W>X	4%	121	5
	PBRM1	Substitution	Nonsynonymous coding	160E>K	6%	67	4
	TSC2	Substitution	Nonsynonymous coding	354Q>R	3%	141	4
Tumor Zone 2	GATA1	Substitution	Nonsynonymous coding	298R>W	2%	188	4
Tumor Zone 3	GNAS	Substitution	Nonsynonymous coding	232R>C	2%	175	4
	MSH6	Substitution	Nonsynonymous coding	922R>Q	4%	92	4

Patient # 9309	Gene	Fold amplification
Tumor Zone 1	GATA2	4.0
	FGFR3	4.3
	NOTCH1	6.2
	HRAS	5.3
	CCND1	7.0
	AKT1	3.1
	IDH2	3.3
	ERBB2	3.4
	GNA11	4.0
	RUNX1	3.6
Tumor Zone 2	FGFR3	3.5
	MYC	3.9
	NOTCH1	4.2
	HRAS	4.3
	CCND1	5.1
	IDH2	3.9
	ERBB2	3.5
	GNA11	3.9
Tumor Zone 3	GATA2	3.0
	FGFR3	3.4
	MYC	3.2
	NOTCH1	3.4
	HRAS	3.7
	CCND1	5.0

Supplementary Table 22. Summary of all mutations and copy number variations identified in AIS samples. Table lists mutated genes, mutations type, amino acid changes, distinct coverage and percentage of mutant reads identified.

Minimally invasive adenocarcinoma

Patient # 2035

Histologic progression within the same lesion	Gene	Mutation Type	Consequence	Amino Acid (protein)	Mutant reads	Distinct Total Reads	Distinct Mutant Reads
**Tumor Zone 1**	**CTNNB1**	Substitution	Nonsynonymous coding	535R>Q	3%	150	4
	**EGFR**	Deletion	In-frame deletion	NA	4%	98	4
	**TP53**	Substitution	Nonsynonymous coding	245G>S	38%	100	38
**Tumor Zone 2**	**EGFR**	Deletion	In-frame deletion	NA	3%	64	2
	**TP53**	Substitution	Nonsynonymous coding	245G>S	34%	77	26
**Tumor Zone 3**	**CREBBP**	Substitution	Nonsynonymous coding	346A>V	2%	204	5
	**EGFR**	Deletion	In-frame deletion	NA	3%	134	4
	**TP53**	Substitution	Nonsynonymous coding	245G>S	46%	194	89
**Tumor Zone 4**	**EGFR**	Deletion	In-frame deletion	NA	3%	134	4
	**TP53**	Substitution	Nonsynonymous coding	245G>S	19%	197	38

Patient # 1061

Histologic progression within the same lesion	Gene	Mutation Type	Consequence	Amino Acid (protein)	Mutant reads	Distinct Total Reads	Distinct Mutant Reads
**Tumor Zone 1**	**BRAF**	Substitution	Nonsynonymous coding	581N>S	3%	202	7
**Tumor Zone 2**	**APC**	Substitution	Nonsynonymous coding	1444Q>R	2%	193	4
	**BRCA2**	Substitution	Nonsynonymous coding	1043A>V	2%	187	4
	**MPL**	Substitution	Nonsynonymous coding	594L>W	10%	220	21
	**PTEN**	Substitution	Nonsynonymous coding	301D>N	2%	230	5
**Tumor Zone 3**	**APC**	Deletion	Frameshift	NA	43%	231	99
	**ASXL1**	Substitution	Nonsynonymous coding	533A>S	39%	673	264
	**ATRX**	Substitution	Nonsynonymous coding	929Q>E	3%	190	5
	**NOTCH3**	Substitution	Nonsynonymous coding	1700D>Y	49%	76	37
	**TP53**	Substitution	Nonsynonymous coding	204E>D	19%	605	115
	**TP53**	Substitution	Nonsynonymous coding	154G>V	55%	556	308
**Tumor Zone 4**	**APC**	Deletion	Frameshift	NA	7%	260	18
	**ASXL1**	Substitution	Nonsynonymous coding	533A>S	16%	319	50
	**NOTCH3**	Substitution	Nonsynonymous coding	1700D>Y	19%	79	15
	**TP53**	Substitution	Nonsynonymous coding	204E>D	11%	356	40
	**TP53**	Substitution	Nonsynonymous coding	154G>V	33%	340	111

Patient # 6610

Histologic progression within the same lesion	Gene	Mutation Type	Consequence	Amino Acid (protein)	Mutant reads	Distinct Total Reads	Distinct Mutant Reads
**Tumor Zone 1**	**EGFR**	Insertion	In-frame insertion	NA	3%	132	4
	**IGF1R**	Substitution	Nonsynonymous coding	983A>T	2%	195	4
	**MED12**	Substitution	Nonsynonymous coding	709E>K	3%	159	4
	**TET2**	Substitution	Nonsense	1050S>X	2%	234	5
	**TP53**	Substitution	Nonsynonymous coding	154G>D	2%	241	5
**Tumor Zone 2**	**CCND1**	Substitution	Nonsynonymous coding	293V>M	3%	147	5
	**EGFR**	Insertion	In-frame insertion	NA	NA	NA	NA
	**FGFR2**	Substitution	Splice site acceptor	NA	2%	174	4
**Tumor Zone 3**	**EGFR**	Insertion	In-frame insertion	NA	3%	209	6
**Tumor Zone 4**	**EGFR**	Insertion	In-frame insertion	NA	2%	376	8
	**NOTCH1**	Deletion	In-frame deletion	NA	4%	114	4

Patient # 6610

Gene	Folds	Type
**CCND1**	3.1	Amplification

Patient # 6992

Histologic progression within the same lesion	Gene	Mutation Type	Consequence	Amino Acid (protein)	Mutant reads	Distinct Total Reads	Distinct Mutant Reads
**Tumor Zone 1**	**ARID1B**	Substitution	Nonsynonymous coding	681G>D	4%	95	4
	**CTNNB1**	Substitution	Splice site acceptor	NA	4%	91	4
	**MED12**	Substitution	Nonsynonymous coding	784R>H	4%	90	4
**Tumor Zone 2**	**EGFR**	Substitution	Nonsynonymous coding	790T>M	7%	623	44
	**EGFR**	Substitution	Nonsynonymous coding	858L>R	8%	617	49
**Tumor Zone 3**	**EGFR**	Substitution	Nonsynonymous coding	790T>M	16%	403	64
	**EGFR**	Substitution	Nonsynonymous coding	858L>R	17%	327	56
**Tumor Zone 4**	**EGFR**	Substitution	Nonsynonymous coding	790T>M	18%	483	89
	**EGFR**	Substitution	Nonsynonymous coding	858L>R	15%	515	76

Patient # 4845

Histologic progression within the same lesion	Gene	Mutation Type	Consequence	Amino Acid (protein)	Mutant reads	Distinct Total Reads	Distinct Mutant Reads
**Tumor Zone 1**	**ATRX**	Substitution	Nonsynonymous coding	183K>E	37%	243	89
	**EGFR**	Substitution	Nonsynonymous coding	858L>R	13%	704	95
**Tumor Zone 2**	**ATRX**	Substitution	Nonsynonymous coding	183K>E	32%	208	67
	**EGFR**	Substitution	Nonsynonymous coding	858L>R	17%	682	118
**Tumor Zone 3**	**ATRX**	Substitution	Nonsynonymous coding	183K>E	32%	318	101
	**EGFR**	Substitution	Nonsynonymous coding	858L>R	15%	668	103
**Tumor Zone 4**	**ATRX**	Substitution	Nonsynonymous coding	183K>E	35%	308	108
	**EGFR**	Substitution	Nonsynonymous coding	858L>R	19%	808	154

Patient # 4845

Gene	Folds	Type
**NOTCH4**	1.8	Amplification

Supplementary Table 23. Summary of all mutations and copy number variations identified in MIA samples. Table lists mutated genes, mutations type, amino acid changes, distinct coverage and percentage of mutant reads identified.

Gene Symbol	location	Genomic position	Amino Acid (protein)	Mutation Type	Sequence Context (Mutation Indicated by "N")	BioRad assay ID
**ATM**	coding area	chr11_107706206-107706206_C_A	p.2455L>M	Substitution	GTGCANTGAAA	dHsaMDS70287642
**ATRX**	coding area	chrX_76824619-76824619_G_C	p.929Q>E	Substitution	ACTCTNCTCTT	dHsaMDS44557415
**BRAF**	coding area	chr7_140127880-140127880_C_A	p.466G>V	Substitution	ATGATNCAGAT	dHsaMDS18827188
**BRAF**	coding area	chr7_140127871-140127871_C_G	p.469G>A	Substitution	CTGTTNCAAAT	dHsaMDS70176053
**CCND1**	coding area	chr11_69175220-69175220_G_A	p.293V>M	Substitution	GGGACNTGGAC	dHsaMDS31524918
**ERBB2**	coding area	chr17_35119948-35119948_G_A	p.243G>S	Substitution	CTGCCNGCTGC	dHsaMDS83096961
**FGFR2**	splice site	chr10_123315211-123315211_G_A	NA	Substitution	GCTCTNCAGAA	dHsaMDS95794691
**IGF1R**	coding area	chr15_97291048-97291048_G_A	p.983A>T	Substitution	TCAGCNCTGCT	dHsaMDS44445826
**IKZF1**	coding area	chr7_50422576-50422576_A_G	p.210Y>C	Substitution	AAGCTNTAAAC	dHsaMDS57366734
**MED12**	coding area	chrX_70261620-70261620_G_A	p.709E>K	Substitution	AGAAGNAGGTG	dHsaMDS18715599
**NRAS**	coding area	chr1_115060268-115060268_C_G	p.13G>R	Substitution	AACACNACCTG	dHsaMDS31636507
**STK11**	splice site	chr19_1171505-1171505_G_T	NA	Substitution	CCGAGNTAGGC	dHsaMDS95906280
**TET2**	coding area	chr4_106377697-106377697_C_A	p.1050S>X	Substitution	CAAATNACAGA	dHsaMDS82985372
**TP53**	coding area	chr17_7519194-7519194_C_T	p.154G>D	Substitution	GGGTGNCGGGC	dHsaMDS57255145

Supplementary Table 24. Summary of ddPCR assays used in this study.
